# Cellular Uptake, Intracellular Fate, and Cytotoxicity of Micro- and Nanoparticulate Nickel Substances in Human Lung Cells

**DOI:** 10.1021/acs.chemrestox.6c00074

**Published:** 2026-08-04

**Authors:** Hanna L. Karlsson, Abishek Arora, Gunilla Herting, Luisa Path, Samuel Buxton, Tara Lyons-Darden, Inger Odnevall

**Affiliations:** † Institute of Environmental Medicine, 27106 Karolinska Institute, SE-17177 Stockholm, Sweden; ‡ Department of Pharmaceutical Biosciences, Uppsala University, SE-751 24 Uppsala, Sweden; § School of Engineering Sciences in Chemistry, Biotechnology and Health, Department of Chemistry, Division of Surface and Corrosion Science, KTH Royal Institute of Technology, SE-100 44 Stockholm, Sweden; ∥ 460764 NiPERA Inc., 2525 Meridian Parkway, Suite 240, Durham, North Carolina 27713, United States; ⊥ AIMES−Center for the Advancement of Integrated Medical and Engineering Sciences at Karolinska Institute, SE-100 44 Stockholm, Sweden

## Abstract

Nickel (Ni) is utilized in a wide range of applications, including the production of austenitic stainless steel, superalloys, rechargeable batteries, catalysts, and foundry products. Historically high exposure to mixed Ni dust has been associated with various health effects, including an increased risk of respiratory cancer. Different chemical forms of Ni exhibit distinct physicochemical properties and biological effects, and the role of these properties, including nanoparticles (NPs) vs microparticles (MPs), in the cellular uptake and fate is not completely understood. This study aims to explore the uptake, intracellular fate, and cytotoxicity of eight well-characterized Ni substances in BEAS-2B cells. Characterization was conducted using scanning electron microscopy, X-ray photoelectron spectroscopy, and dynamic light scattering. Uptake in BEAS-2B cells was investigated using transmission electron microscopy, Newport Green staining in combination with super-resolution microscopy, and atomic absorption spectroscopy. The characterization showed that the Ni substances differed in primary size but were all highly agglomerated under the conditions tested, with Ni_3_S_2_ having the largest size. At 48 h and based on the same mass of added Ni, Ni_3_S_2_ was the most cytotoxic, followed by the water-soluble salt NiSO_4_·6H_2_O and Ni metal NPs with a primary size of 80 nm (Ni80), while nickel oxide (NiO) NPs were noncytotoxic under the tested conditions. Biodissolution (released Ni per mass Ni added) in cell culture medium varied substantially across substances and generally increased with time. At 48 h, Ni biodissolution followed this order: Ni_3_S_2_ (55%) > Ni80 (9%) > NiO MPs (4%) > Ni 20 nm and NiO 80 nm (3%), > NiO 20 nm (2%) > Ni MPs (0.3%). All sparingly soluble Ni substances were clearly observable in intracellular membrane-bound structures in BEAS-2B cells. A key observation from the quantitative uptake was the distinctly lower uptake of Ni ions from the water-soluble NiSO_4_·6H_2_O compared to the sparingly soluble Ni substances. There was thus no clear correlation between apparent uptake and cytotoxicity. After fractionation into cytosolic, nuclear, and particulate fractions, followed by analysis of dissolved Ni from the particles within the cells, the three substances showing the highest amount of Ni per added Ni mass (indicating both uptake and intracellular Ni ion release) in both cytosol and nucleus were Ni20 NPs, Ni MPs, and Ni_3_S_2_. Super-resolution microscopy indicated increased Newport Green fluorescence, suggestive of intracellular Ni accumulation, predominantly in cells exposed to Ni MPs and Ni_3_S_2_. However, the findings were not sufficiently robust to allow firm conclusions. These findings highlight the complex interplay between biodissolution, cellular uptake and cytotoxicity of Ni substances.

## Graphical abstract

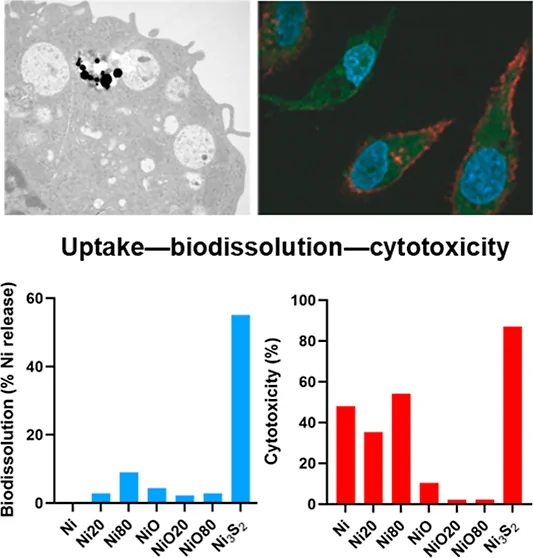

## Introduction

Nickel (Ni) is utilized in a wide range of applications due to its numerous beneficial properties. According to the US Geological Survey, approximately 65% of the Ni consumed in the Western World is utilized in the production of austenitic stainless steels, known for their high corrosion resistance and formability, and 12% is dedicated to the creation of superalloys, which are essential for aerospace and power generation applications.^1^ The remaining 23% of the Ni consumption is allocated to various applications such as an alloying metal and use in rechargeable batteries, as catalysts, foundry products, and for plating applications.^1^ High historical industrial exposure to mixed Ni dust, with coexposures to other organic and inorganic substances, has been associated with various health effects, including respiratory cancer.^2^ Consequently, the International Agency for Research on Cancer has classified Ni compounds as carcinogenic to humans (Group 1), while Ni metal is classified as possibly carcinogenic to humans (Group 2B).^3,4^ Inhalation exposure to Ni compounds has also been associated with a range of respiratory conditions, including irritation, inflammation, bronchitis, pulmonary fibrosis, asthma, and pulmonary edema.^5^ As described by IARC[Bibr ref4] (2012), inhalation exposures occur in both Ni-producing industries (such as mining and smelting) and Ni-using industries (including for example alloy- and stainless-steel manufacturing, electroplating, welding, and cutting). Recently, the evidence supporting causal relationships between exposure to Ni in ambient particulate matter (PM) and respiratory or cardiovascular outcomes was evaluated.^6^ The review included 38 studies conducted on human populations, published between 2012 and 2022, suggesting that exposure to Ni in ambient PM was statistically significantly associated with several outcomes including respiratory mortality, respiratory emergency hospital visits, asthma, and cardiovascular mortality. Due to methodological limitations, however, the authors still did not consider the literature to provide “adequate support” for a causal relationship.

The various chemical forms of Ni exhibit different physicochemical properties and biological effects. For example, in vivo acute oral toxicity (rats) is well predicted by in vitro gastric bioaccessibility (% Ni release).^7^ Conversely, long-term inhalation bioassay studies on rats have demonstrated that only poorly soluble compounds, such as nickel oxide (NiO) and nickel subsulfide (Ni_3_S_2_) particles, induce lung tumors, whereas soluble Ni compounds and insoluble nickel metal did not exhibit any carcinogenic potential.^8–12^ The discrepancy for soluble Ni compounds has been attributed to the rapid clearance and limited cellular uptake of soluble Ni compounds. Several studies conducted in the 1980s demonstrated significant differences in the cellular uptake between various Ni compounds. Notably, crystalline Ni_3_S_2_, a potent carcinogen, was taken up by cells to a much greater extent than amorphous NiS, which was considered noncarcinogenic.^13,14^ It should be noted, however, that particle uptake appears to be cell-type dependent. The differences in the uptake were, for example, more pronounced in Syrian hamster embryo cells compared to Chinese hamster ovary (CHO) cells. One proposed explanation for this disparity involves surface charge characteristics. Carcinogenic particles such as crystalline NiS, Ni_3_S_2_, and NiO exhibit strongly negative zeta potentials in distilled water and are readily internalized by cells. In contrast, the noncarcinogenic amorphous NiS, which shows limited phagocytosis, possesses a slightly positive surface charge under similar conditions. However, since the surface charge is highly particle- and solution dependent (e.g., due to differences in ionic strength), findings in distilled water are considerably different compared to cell conditions.^15^ Another important factor is size; Ni_3_S_2_ particles between 1.8 and 4 μm were taken up more frequently than those between 4 and 5 μm, and particles exceeding 5 μm were rarely taken up.^14^ Although the uptake process was initially described as phagocytosis, subsequent studies have suggested that it may instead involve macropinocytosis and/or clathrin-mediated endocytosis.^5^ Soluble Ni enter the cell via the divalent metal transporter 1 and possibly also through calcium channels.^16,17^ While the role of particle size in the uptake and toxicity is clearly important, its investigation is often complicated by severe agglomeration in biological media as a result of strong van der Waals forces, which can obscure size-dependent effects.^18^

A model has been suggested that links the respiratory carcinogenic potential to the bioavailability of Ni (as ions, labile or strongly bonded complexes) at nuclear sites within respiratory target cells. This “nickel bioavailability model” suggest that the Ni species with the highest potential to induce tumors are those that (a) are insoluble enough to persist in the lungs as particles, (b) can efficiently enter epithelial cells via phagocytosis, and (c) release Ni ions within phagosomes, which can then interact with DNA and proteins in the nucleus.^13,19,20^

The influence of various physicochemical properties of Ni compounds—such as particle size, surface composition, surface charge, and biodissolution—on the cellular uptake and intracellular fate remains incompletely understood. Since surface charge measurements are highly dependent on the surrounding medium,^18,21^ the zeta potential of particles measured in cell culture medium is determined primarily by the adsorbed biomolecular corona rather than by the underlying particle surface. Consequently, under these conditions, zeta-potential measurements provide limited information on how intrinsic particle surface properties influence cellular uptake and intracellular fate.^18^ Previous research has demonstrated that multiple factors can significantly affect the extent of particulate uptake. This complexity underscores the importance of thoroughly characterizing uptake when assessing toxicity in the context of various Ni compounds.^5^ In our previous studies, we investigated the uptake of different Ni substances in human lung epithelial cells. For instance, in A549 cells, both Ni and NiO were taken up by the cells after 4 h of exposure. Notably, Ni—both nano- and microscale—exhibited slightly higher uptake compared to NiO particles.^22^ Further experiments in BEAS-2B cells revealed comparable uptake levels for Ni and NiO nanoparticles (NPs), while soluble ionic Ni (from NiCl_2_·H_2_O) showed significantly lower cellular association.^23,24^ These findings suggest, similar to previous observations, that biodissolution, chemical form, and cell type all play a critical role in cellular interactions and should be carefully considered in future toxicological assessments. A key objective is to better understand the role of particle size, as well as to explore potential changes in Ni localization to the nuclear compartment. This is particularly important given that nuclear delivery has been proposed as a critical factor underlying the high carcinogenic potential of Ni_3_S_2_, in contrast to the lack of carcinogenicity observed in rats exposed to soluble Ni.^20^

The overall aim of this study was to investigate the cellular uptake, intracellular fate, biodissolution, and cytotoxicity of well-characterized Ni substances in BEAS-2B cells, which represent target cells of Ni respiratory toxicity. Specifically, eight distinct Ni substances were examined, including Ni metal and Ni oxide (NiO) particles in both micro- and nanoscale forms, Ni subsulfide (Ni_3_S_2_), and the water-soluble salt Ni sulfate hexahydrate (NiSO_4_·6H_2_O). All exposures were based on comparing the same added Ni mass. Particle characterization was conducted using scanning electron microscopy (SEM), X-ray photoelectron spectroscopy (XPS), and dynamic light scattering (DLS). The cellular uptake was assessed using transmission electron microscopy (TEM), Newport Green staining in combination with super-resolution microscopy, and quantitatively determined by graphite furnace atomic absorption spectroscopy (GF-AAS). Additionally, subcellular fractionation of cytosolic and nuclear compartments,^25^ followed by AAS, was performed to evaluate the intracellular distribution of Ni.

## Methods

### Ni Substances

In total, eight Ni substances were investigated—Ni metal micron-sized powder (Ni microparticle (MP), CAS# 7440-02-0, Sumitomo, Tokyo, Japan), Ni metal nanopowders (80 nm—Ni80 NP and 20 nm—Ni20 NP, Miyou, Suzhou, China), Ni oxide black micron-sized powder (NiO MP, CAS# 1313-99-1, Umicore, Brussels, Belgium), Ni oxide nanopowders (80 nm—NiO80 and 20 nm—NiO20, Miyou, Suzhou, China), micron-sized Ni subsulfide (Ni_3_S_2_, CAS# 12035-72-2, Vale Base Metals). Soluble Ni sulfate hexahydrate (NiSO_4_·6H_2_O, CAS# 10101-97-0, Sigma-Aldrich, USA) was included for comparison.

The investigated particle loading was based on the mass of Ni and not on the particle mass to enable comparisons of the different Ni-based particles from their total Ni content perspective. As a result, the exposures were conducted with different powder mass. For calculations of the particle loading in relation to the Ni content in the different powders, the molecular weight of each powder was used: 58.69 g/mol for Ni MPs, Ni80, and Ni20 NPs; 74.69 g/mol for NiO MPs, NiO80, and NiO20 NPs; 240.25 g/mol for Ni_3_S_2_, and 262.85 g/mol for NiSO_4_·6H_2_O. In terms of mass for the substances, 100 μg Ni/mL corresponds to 127 μg substance/mL for NiO, 409 μg substance/mL for Ni_3_S_2_, and 448 μg substance/mL for NiSO_4_·6H_2_O.

### Characterization of Particle Morphology, Size, and Surface Composition

SEM, using a FEI XL30 ESEM instrument, was used to determine particle morphology, size, and extent of aggregation of assemblies of particles of the different Ni substances placed on carbon tape. XPS analysis was conducted to determine the composition of the outermost 5–10 nm surfaces of the Ni substances. Spectra were recorded for two assemblies of particles mounted on carbon tape using a Kratos AXIS UltraDLD X-ray photoelectron spectrometer (Kratos Analytical, Manchester, UK) with a monochromatic Al X-ray source (1486.6 eV) operating at 300 W. Both wide-scan and high-resolution spectra (20 eV) were collected for C 1s, O 1s, and Ni 2p to evaluate surface oxidation. All binding energies were calibrated using the adventitious carbon (C 1s) peak at 285.0 eV. The Ni 2p XPS spectra were separated into individual component peaks (peak deconvolution) by constrained peak fitting using Gaussian–Lorentzian line shapes and an appropriate background subtraction to resolve the contributions from different nickel chemical states, spin–orbit components, and satellite features.^25,26^

Particle stability and hydrodynamic particle sizes were investigated for all poorly soluble Ni substances with DLS (Malvern Zetasizer nano ZS, UK) using standard UV cuvettes (Kartell S.p.A. Noviglio, Italy). Standard latex samples (3000 Series Nanosphere Size Standards, 0.06 ± 0.004 μm, Thermo Scientific) were tested to verify the precision of the measurements. All measurements were performed at 37.0 ± 0.1 °C. Since the objective was to evaluate particle stability under conditions representative of the toxicity study while minimizing sonication-induced modifications of particle surface properties and metal migration, the ultrasonication duration was restricted to a minimum (1 min).^18,27^ This approach is also reflective of real-world exposures. Due to too low particle intensity, the measurements could not be conducted at a particle loading of 0.1 mg/mL, therefore the concentration was increased to 0.5 mg/mL. A stock solution of 1 mg Ni/mL was prepared for each of the Ni substances in BEGM (Lonza, Switzerland), preheated to 37.0 ± 0.1 °C. The stock solution was water bath ultrasonicated for 1 min then diluted to 0.5 mg/mL by pipetting 1 mL of the stock solution into a cuvette and adding 1 mL of BEGM. The stock solution was vortexed for 1 s before collecting each 1 mL for analysis.

### Sample Preparation for Biodissolution

Stock suspensions (1 mg Ni/mL) of each Ni substance were prepared in the BEGM cell medium followed by ultrasonication for 1 min (conditions representative of the toxicity study) at room temperature using a water bath. For the soluble Ni salt, NiSO_4_·6H_2_O, 1 mg Ni/mL of stock solution in water was prepared without any sonication. This salt was included as a reference solution with mainly free Ni ions. 1 mL of the stock suspension was pipetted to a new vessel and diluted with 9 mL of fresh medium to generate a Ni loading of 0.1 mg/mL (theoretically 1 mg of Ni in 10 mL of medium) for each Ni substance solution. This setup intentionally deviates from standard biodissolution testing, which typically involves direct preparation of the target particle concentrations. Instead, the procedure mimics that used in the cellular toxicity studies, including stock solution preparation, sonication, and serial dilutions as described below and in the cited studies.^18,28^ Triplicate samples of each particle type were exposed under dark conditions at 37 °C for 30 min, 4 h, 24 h, 48 h, and 72 h in the BEGM cell media using gentle bilinear agitation (25 rpm). Parallel exposure time periods were investigated for the water-soluble Ni salt in BEGM to monitor any possible sedimentation effects or losses during the following separation due to Ni complexation with time. In addition, one blank sample (cell medium without any addition of particles) was incubated together with the triplicates for each time period to determine background concentrations, if any. After exposure, 9 mL of the samples was separated from the powder particles by using alumina-based syringe filters with a pore size of 20 nm (Anotop 25, Whatman, USA). The remaining 1 mL was collected and dissolved in diluted aqua regia (2.5 vol % HCl and 6.5 vol % HNO_3_, pH < 0.1) for total particle loading analysis (transferred dose). All solution samples were stored in new vessels and analyzed for total Ni concentrations by means of flame or GF-AAS as described below.

### AAS for Biodissolution Testing, Ni Cell Uptake, and Nuclei/Cytosolic Fraction Analysis

AAS using a PerkinElmer atomic absorption spectrometer PinAAcle 900T was used to determine total Ni concentrations in the solution samples. Blank samples (BEGM without particles) were prepared and measured for each exposure condition and subtracted from the measurement of the different Ni substances to remove background contamination, if any. All samples were acidified to a pH below 2 (by using 65% HNO_3_) to enable the total Ni concentration analysis.

The total Ni analysis of the cell exposures and the nuclei/cytosolic fractions was performed using flame and graphite furnace modes, respectively, and required digestion of the cell nuclei as well as potentially present particles to enable total Ni analysis. An acid mix of 10 vol % HCl and 10 vol % HNO_3_ was added to the nuclei/cytosolic fractions samples at a ratio of 1 part sample to 4 parts acid mix. The samples were vortexed 1 min at room temperature and left for 1 week to ensure total dissolution. Blank samples were prepared following the same procedure. Calibration was performed using standards with the known concentrations (0, 10, 30, 100 μg Ni/L), and quality control samples were run every 6 samples. The limit of detection was 0.6 μg Ni/L, and the limit of quantification was 1.5 μg Ni/L. The total sample volume of the nuclei/cytosolic fraction analysis for each Ni substance was 300 μL (5 replicate samples). To enable analysis of such small volumes using GF-AAS required the modification of sample cups and careful adjustments of the instrument autosampling pipetting procedure to ensure representative solution samples for the analysis.

### Cell Culturing and Exposure

Monocultures of the human bronchial epithelial cell line BEAS-2B at passages 15–25 were used in this study. Cells were maintained in precoated culture flasks (75 cm^2^) in a standard humidified incubator (5% CO_2_, 37 °C) and split every 3–4 days. Coating was made from pure BEGM medium (Lonza, Switzerland) supplemented with 0.01 mg/mL fibronectin, 0.03 mg/mL bovine collagen type I, 0.01 mg/mL bovine serum albumin, and 0.2% PEST. Cells were cultured in supplemented BEGM medium. For exposure preparation, all powders were weighed in glass vials based on their respective Ni mass. The water-soluble salt, NiSO_4_·6H_2_O, was dissolved in water and stored at 4 °C prior to exposure preparation. Immediately before exposure, stock suspensions of 1 mg/mL Ni (for water insoluble Ni samples) were prepared freshly in cell medium. Stock suspensions were vortexed at highest power for 5 s followed by ultrasonication for 1 min. These stock suspensions were then immediately diluted in cell media to the final concentrations.

### Cytotoxicity Testing

Alamar blue assay was performed to assess the cytotoxicity of the various Ni substances after 24 h, 48 h, and 72 h exposure of BEAS-2B cells. BEAS-2B cells were first seeded in 96-well plates; 10,000 cells/well for 24 h and 5000 cells/well for 48 and 72 h. Cells were allowed to attach for 24 h. At exposure, the cell medium was removed and 100 μL/well of exposure medium (particle suspensions) was added. Cell medium was used as negative control and 10% dimethyl sulfoxide served as positive control. The exposure concentrations were based on the same mass of Ni for all substances and ranged between 5 and 150 μg Ni/mL. In terms of mass for the substances, this corresponds to ×1.27 for NiO (6–191 μg/mL), ×4.09 for Ni_3_S_2_ (20–614 μg/mL), and ×4.48 for NiSO_4_·6H_2_O (22–672 μg/mL). After exposure in the incubator, the exposure medium was removed and 10% alamarBlue (Thermo Fisher Scientific) in fresh medium was added for 2 more hours of incubation. The plates were read at 540 nm/590 nm excitation/emission wavelength (Tecan Infinite F 200 Pro, Software: Magellan 7.2). To adjust for background fluorescence, blank wells containing only medium were subtracted from all fluorescence values. The fluorescence values were normalized to the mean fluorescence of the negative control, which represented 100% cell viability. Three independent experiments were performed with three technical replicates for each concentration.

### TEM Imaging for Qualitative Analysis of the Uptake

BEAS-2B cells were seeded in 6-well plates at concentrations of 300,000 cells/well for 24 h and 150,000 cells/well for 48 and 72 h. Cells were allowed to attach for 24 h. At the time of exposure, the cell medium was removed and 3 mL/well of particle suspensions at a concentration of 5 μg/mL were added. Cell medium was used as negative control. After 48 h exposure, cells were washed twice with phosphate-buffered saline (PBS) and trypsinized. 1 mL cell medium was added to each well, and samples were transferred to tubes and centrifuged for 4 min at 2000 rpm. The supernatant was removed, and 1 mL of 2.5% glutaraldehyde (Ladd Research, USA) in 0.1 M phosphate buffer was added to each tube. Samples were stored at 4 °C until analysis using Hitachi HT7700 + 2k × 2k Veleta CCD camera.

### Preparation of Cells for Quantitative Analysis of the Ni Uptake Using AAS

The total cellular uptake of Ni was initially assessed for three time points (24 h, 48 h, 72 h) and was then focused on 4 and 24 h. In addition, 4 h of exposure followed by gentle PBS wash and 20 h recovery was also included. BEAS-2B cells were seeded in 24-well plates at concentrations of 60,000 cells/well and were allowed to attach for 24 h.

Next, cells were exposed to 5 μg Ni/mL and 10 μg Ni/mL in 500 μL (corresponding to 2.5 and 5 μg Ni, respectively) of the Ni substances and collected after 4 and 24 h each. Samples, denoted A and B, were collected according to below steps:

A (wash, Ni not in cells): At each time point, the exposure media (500 μL) was collected in a 2 mL microfuge tube, followed by the collection of three subsequent and gentle PBS washes (250 μL each). To wash the wells after the cells were harvested (in “B”), 500 μL of concentrated HNO_3_ was added and incubated at room temperature for 1 h. This was then collected and added to the 2 mL microfuge tube from Part A (final volume: 1750 μL).

B (Ni in, or firmly attached to cells): After the three washes with PBS in “A” above, 50 μL of trypsin was added to each well, followed by incubation at 5% CO_2_ and 37 °C for 5 min. To this, 210 μL of BEGM medium was added and the cells were harvested in suspension and transferred to a 1.5 mL microfuge tube. 10 μL of each cell suspension was collected in a 0.5 mL microfuge tube for counting. Cells were counted with erythrosine b and an automated cell counter (Thermo, Countess II). Total cell counts, live cell counts, and percentage viability were noted.

### Preparation of Cells and Cell-Fractioning for Analysis of Dissolved Ni in Cytosol and Nuclei

BEAS-2B cells were seeded in 6-well plates at a concentration of 150,000 cells/well and were allowed to attach for 24 h. Next, cells were exposed to 5 μg Ni/mL (3 mL/well) for 48 h. After exposure, cells were washed twice with PBS and trypsinized. A PBS/phosphatase inhibitor was added to each well and cells were transferred to tubes. Cells were counted, centrifuged (5 min, 200*g*, 4C), and supernatants were removed. Five technical replicates were performed for each compound.

The Nuclear Extract Kit (Active Motif) was used to separate the nuclear fraction from the cytosolic fraction. Briefly, cells were allowed to swell in hypotonic buffer for 15 min and then lysed by vortexing in the presence of detergent. Cells were centrifuged (30 s, 14,000*g*, 4 °C), and supernatants (cytoplasmic fraction) were transferred to fresh tubes and stored at 4 °C until analysis. Ni that has dissolved intracellularly (including within lysosomes) but has not been translocated to the nucleus is expected to be primarily recovered in this fraction. To obtain the nuclear fraction, the remaining pellets were resuspended in complete lysis buffer, incubated on ice on a rocking platform (30 min, 150 rpm), and vortexed at highest setting before centrifugation (10 min, 14,000*g*, 4 °C). Supernatants, the nuclear fractions, were transferred to fresh tubes and kept at 4 °C until analysis. Ni dissolved within the cells and subsequently translocated to the nucleus is expected to be found in this fraction. Note that the remaining pellet will contain the nondissolved Ni particles taken up by the cells (including particles in lysosomes). Five replicate samples of each fraction were analyzed with GF-AAS. The mass of Ni in each fraction was then divided by the number of cells and expressed as pg/1000 cells.

### Newport Green Staining of Ni Ions in Cells Using Super-Resolution Microscopy

Super-resolution microscopy was used to determine the distribution of Ni ions in BEAS-2B cells following exposure to the Ni substances. Ni ions (and or weak ligands) were visualized using the Newport Green DCF (Invitrogen, N7991) staining that binds to transition-metal ions (Ni, Zn, Mn, Fe, Co, Cu­(I), Cu­(II), and Cd) and thereby exhibits fluorescence with an excitation and emission spectra of 505 and 535 nm, respectively. The nucleus was stained with Hoechst 33258 and the actin cytoskeleton with the Alexa Fluor 647 phalloidin probe. This enabled the visual demarcation of the nuclear and cytoplasmic compartments of the cells. Super-resolution microscopy was conducted on a Zeiss LSM 980 with an Airyscan 2 detector, and the images were processed using the Zeiss ZEN Blue software suite.

BEAS-2B cells were seeded in precoated 24 well plates with coverslips (14 mm) at a seeding density of 0.1 × 10^6^ cells/well in 600 μL/well of BEGM medium. Exposures (50 μg Ni/mL) were performed the next day after the seeding. At least two wells were prepared per exposure, along with two wells of negative controls (untreated cells). The exposure was 24 h, after which the cells were fixed with 4% PFA for 20 min (200 μL/well). The PFA was then aspirated, followed by two quick washes with 1X-TBS and two washes for 10 min each with 1X-TBS. At this point, the cells were left in the last wash and stored at 4 °C, until staining was performed. Staining buffer was prepared by adding 0.1% Triton X-100 in 1X-TBS. To this, 1:500 Hoechst 33342 (Thermo, H3570), 1:1000 Phalloidin (Thermo, A30107) and 1:100 dilution of Newport Green DCF were added. After addition, the samples were incubated at room temperature for 1 h. The staining buffer was then aspirated, followed by three quick washes with 1X-TBS and two washes for 10 min each with 1X-TBS. The coverslips were removed, air-dried, and mounted using mounting medium (Dako). The stained and mounted slides were stored flat at 4 °C overnight, followed by transfer to an opaque slide box for long-term storage at 4 °C. Images were acquired using the 10X air-objective and 63X oil-objective in the snapshot mode for both and Z-stack mode for the latter. Laser intensities were optimized and consistent across all acquired images, and staining was performed using a master-mix to rule out any potential deviations. Cell status was visually observed prior to the staining, to maintain uniform conditions.

## Statistics

Statistical analyses were performed using GraphPad Prism (version 10.1.2). Differences in the cellular uptake among the various Ni substances were assessed using ordinary one-way ANOVA followed by Tukey’s multiple comparisons test. Differences in the intracellular Ni content between the 4 h exposure and the 4 h + 20 h recovery period within each Ni substance were analyzed using two-way ANOVA followed by Šídák’s correction for multiple comparisons. Correlation between cytotoxicity at 100 μg Ni/mL and Ni release at 100 μg Ni/mL was assessed using Spearman’s rank correlation coefficient. A *p*-value <0.05 was considered statistically significant.

## Results

### Particle Morphology, Size, and Surface Composition Characterization

SEM images of the different Ni-based substances show extensive particle agglomeration under dry conditions, Figure 1. This is expected due to the high surface energy and exceptionally strong attractive van der Waals forces of nonfunctionalized metal particles, both in air and solution.^15,18^ Even though highly aggregated, most micrometer- and nanometer-sized Ni metal particles showed a spherical morphology. Large micrometer-sized aggregates of rectangular prism-shaped particles were also observed for the NiO substances, whereas the Ni_3_S_2_ substance consisted of micrometer-sized lump particles of irregular morphology.

**Figure 1:**
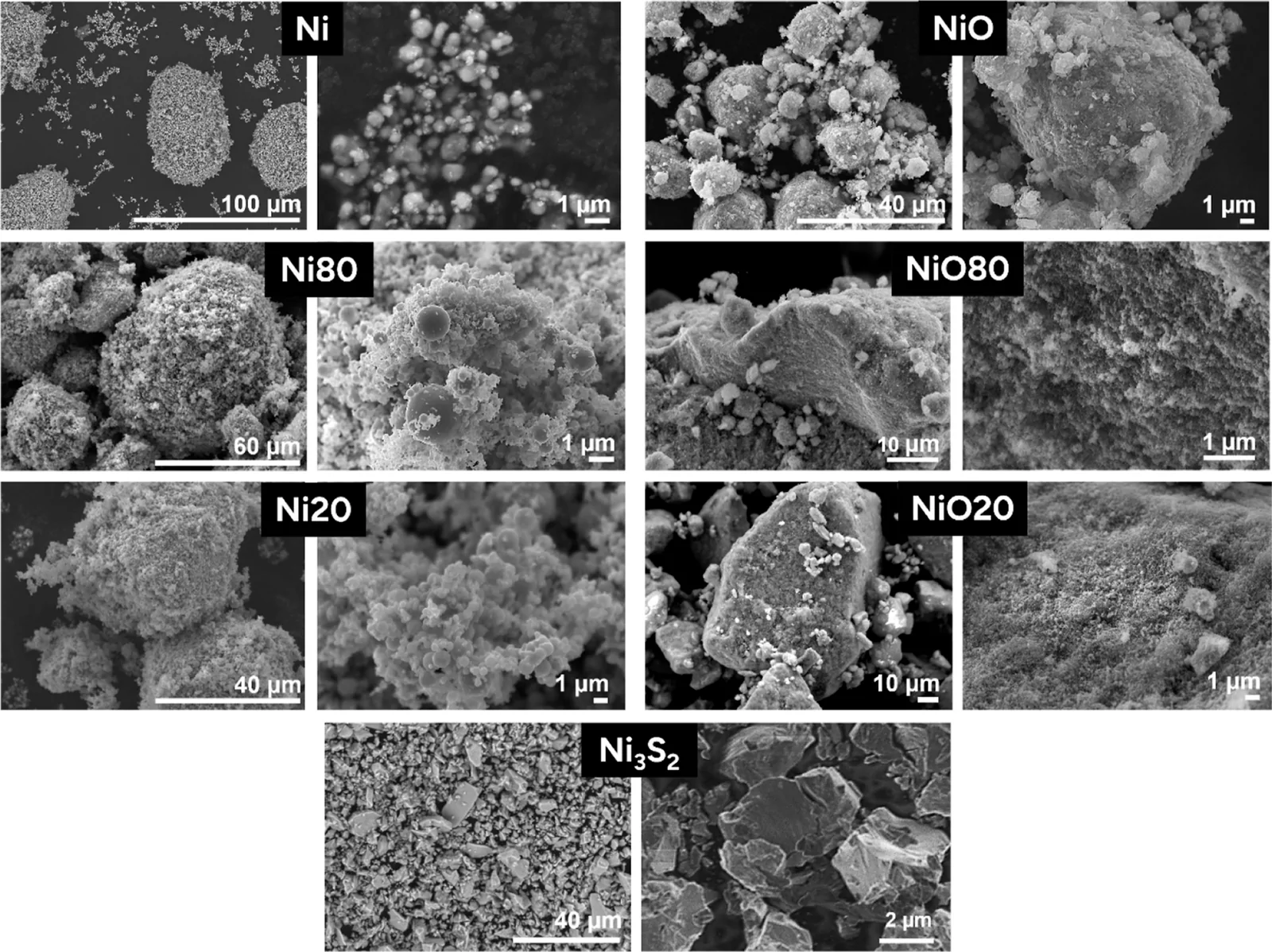
SEM images of the different Ni substances.

Extensive aggregation of the Ni substances observed under dry conditions, Figure 1, was also evident from the DLS investigation upon dispersion in BEGM cell medium, Table 1, measurements conducted to reflect the conditions at cell exposure. All particles were highly polydisperse (PDI ≥ 0.4), which reflects extensive aggregation, broad size distributions, and mixed particle size populations. The results are therefore presented as *Z*-average values, illustrating the intensity-weighed harmonic mean diameters of the entire particle/aggregate size distribution showing micrometer-sized aggregates, rather than nanosized agglomerates for all substances in suspension. This reduces evidently the accessible surface area of the NPs, though this area largely depends on the packing density and porosity of the aggregate. The actual surface areas of the Ni substances exposed to solution may be very different from the indicated *Z*-average size distributions. All particles were micrometer sized, with the irregularly shaped (lump) Ni_3_S_2_ substance (Figure 1) showing the largest size, and the NiO MPs the smallest aggregates.

**Table 1:** Aggregate Size Distribution of All Ni Substances, Except NiSO_4_·6H_2_O in Suspension, Described as *Z*-Average Values Illustrating the Intensity-Weighed Harmonic Mean Diameters Measured with DLS^a^

(μm)	Ni	Ni80	Ni20	NiO	NiO80	NiO20	Ni_3_S_2_
*Z* average	3.4 ± 0.4	4.2 ± 0.6	3.9 ± 0.4	1.4 ± 0.3	4.3 ± 2.1	3.8 ± 1.3	7.1 ± 0.2^b^
PDI	1.0 ± 0.0	0.52 ± 0.42	0.97 ± 0.06	0.56 ± 0.10	0.57 ± 0.32	0.40 ± 0.16	1.0 ± 0.0

a The measurements were done at a concentration of 0.5 mg Ni/mL in BEGM media after 1 min sonication.

b Poor fit due to nonspherical lump particles, PDI—polydispersive index.

Surface compositional analysis by means of XPS of the outermost surface oxide on the metal Ni and NiO particles are presented in Figure 2. Peak deconvolution of the metal Ni 2p_3/2_ particle spectra clearly revealed the predominant presence of NiO (853.9 ± 0.03 eV) with smaller amounts of Ni­(OH)_2_ (855.8 ± 0.10 eV) for all metal Ni substances. Deconvolution of the spectra of the NiO particles showed a Ni 2p_3/2_ peak at 853.5 ± 0.03 eV, corresponding to NiO. The Ni metal signal was detected for all Ni metal particles, with the strongest contribution from Ni80 NPs (Ni_met_/Ni_total_ atomic ratio: 25.1 ± 0.86), followed by Ni20 NPs (18.8 ± 2.59) and Ni MPs (7.50 ± 1.80), indicating Ni80 NPs to have the thinnest surface oxide, followed by the Ni20 NPs and the Ni MPs, all in the nanometer range (<5 nm). The relative atomic fractions of Ni­(OH)_2_ compared to NiO for the same particles were calculated to 11.5 ± 2.7% for the Ni80 NPs, 5.5 ± 0.8% for the Ni20 NPs, and 3.8 ± 0.2% for the Ni MPs.

**Figure 2:**
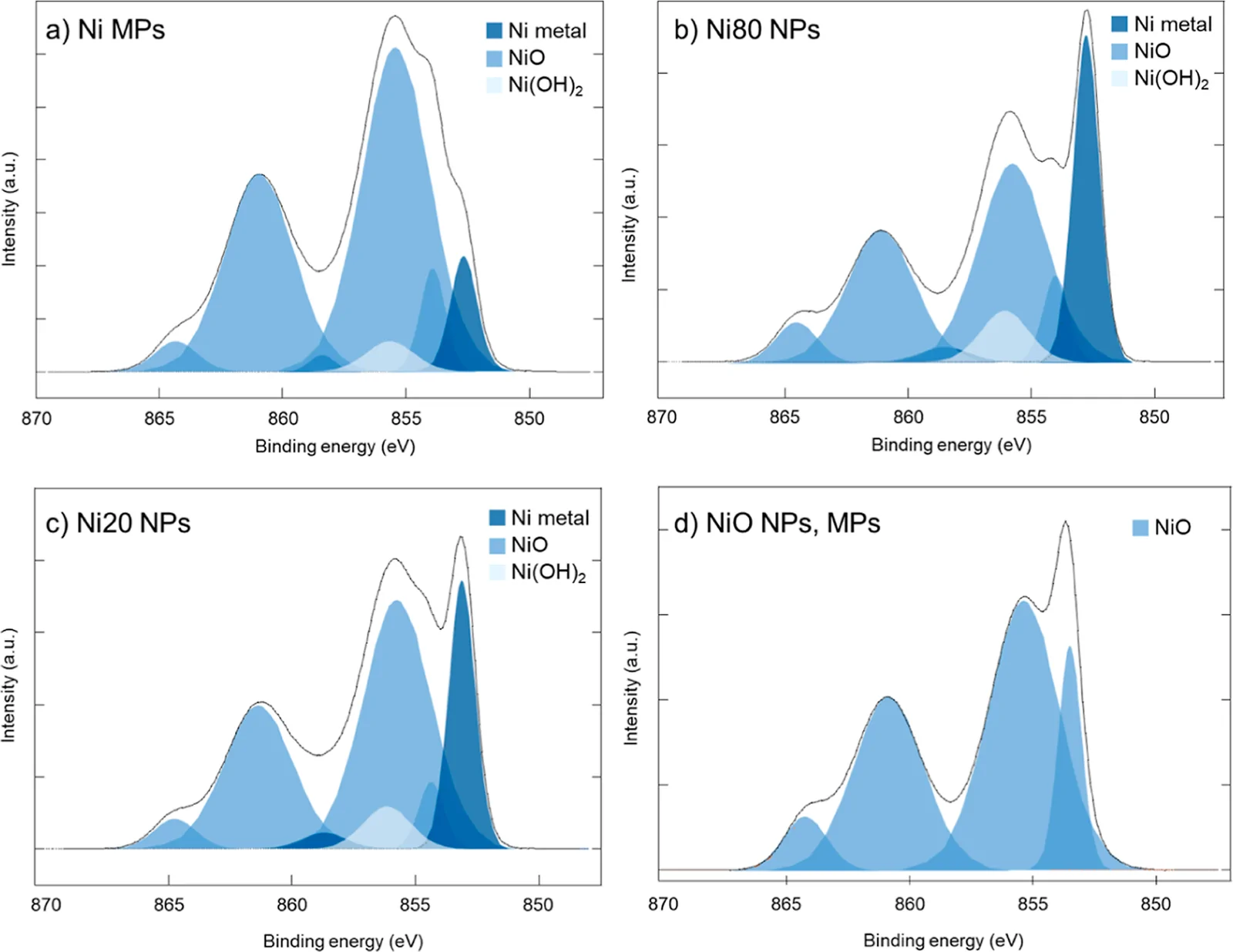
Peak deconvolution of Ni 2p_3/2_ XPS spectra of the nano and micron-sized Ni metal substances (a–c) and the NiO substances (d) assessing the composition of the outermost surface oxide (top 5–10 nm).

### Biodissolution in Cell Medium

In a recent paper, we have shown the transferred dose of the same Ni substances as in this study to be considerably lower than the nominal dose because of extensive aggregation and sedimentation, emphasizing the necessity of measuring dose samples for all exposure conditions and time points.^18^ The kinetic biodissolution results presented in Figure 3 are therefore expressed as the extent of released Ni per mass of Ni added (rather than particle mass) for each Ni substance. The exposed particle mass was higher for the Ni20 NPs and Ni80 NPs compared to the Ni MPs, and similarly higher for the NiO NPs, Ni MPs, and Ni_3_S_2_. It should be noted that this experimental setup intentionally deviates from standard biodissolution testing^21^ where particle dispersions are typically prepared directly at the target concentrations without successive dilutions. The setup aimed to replicate the cell toxicity procedures, including stock suspension preparation, sonication, and serial dilutions (see Section Methods and ref 18). The extent of released Ni varied considerably with time and between the Ni substances. As expected, the water-soluble NiSO_4_·6H_2_O salt almost completely remained in its dissolved state, independent of time. For the other substances, the Ni metal MPs were the least soluble (<1%) and the Ni_3_S_2_ (62%) the most soluble after 72 h. An increased extent of Ni biodissolution was generally observed for all powders with time. Among the Ni metal powders, the release of Ni was the highest from the Ni80 NPs after 72 h (14%) compared to the Ni20 NPs (5%) and the Ni MPs (<1%).

**Figure 3:**
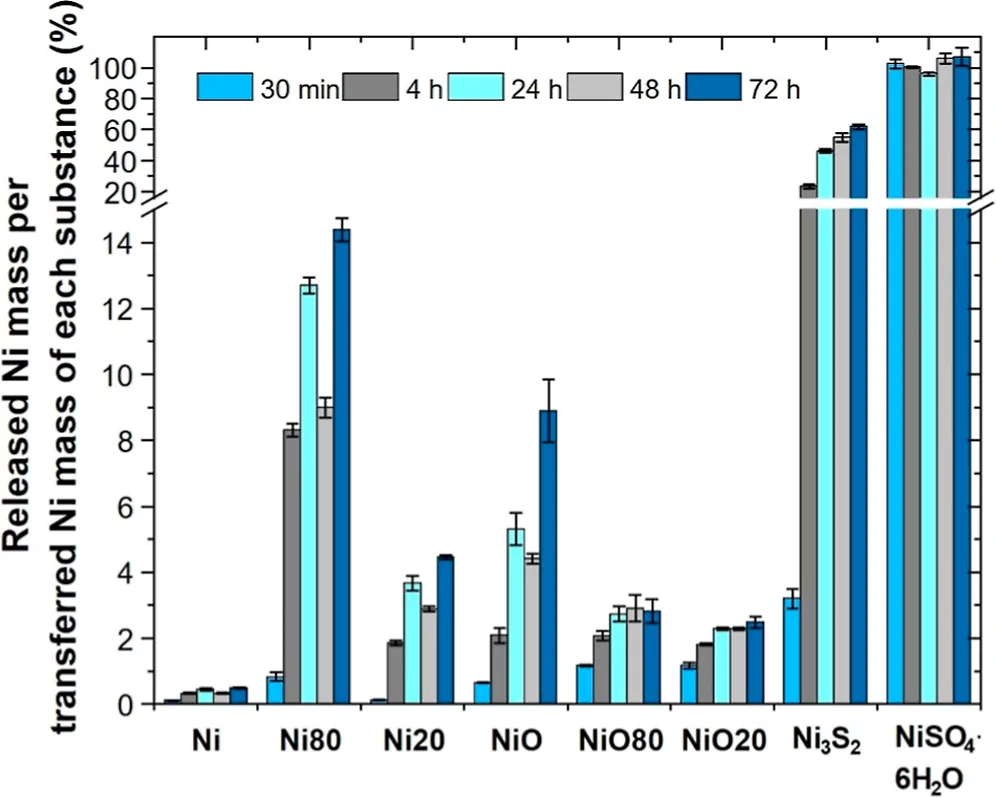
Kinetic biodissolution results presented as released mass of Ni per transferred Ni mass (nominally 100 μg Ni/mL) from the different Ni substances in BEGM cell medium. Parallel measurements of the completely dissolved NiSO_4_·6H_2_O dispersion (no particles) are included assessing eventual sedimentation of Ni complexes in the cell medium with time.

### Cytotoxicity of the Various Ni Substances

BEAS-2B cells were exposed to the various Ni substances, and cytotoxicity was analyzed following 24 h, 48 h, and 72 h of exposure. The results are shown in Figure 4. The results show that the exposure to the soluble Ni salt (NiSO_4_·6H_2_O) and particles of Ni_3_S_2_ were the only conditions that caused cytotoxic effects at the first time point (24 h). Ni20 caused a small but significant increase in viability (metabolic activity) at this time point. All Ni substances, except the NiO80 and the NiO20 NPs, caused cytotoxicity at 48 h, with the third highest cytotoxicity (based on added Ni mass) observed with Ni80. This is in line with the biodissolution results showing the highest dissolution for Ni_3_S_2_ (55%) at this time point followed by Ni80 (≈15%) and NiO MPs (≈9%), whereas no direct correlation between biodissolution and cytotoxicity could be observed for the other Ni substances showing less than 5% dissolution. The calculated EC50 values (μg Ni/mL) after 48 h, from most toxic (low EC50) to least toxic, were 3.5 (Ni_3_S_2_), 39 (Ni80 NPs), 63 (NiSO_4_·6H_2_O), 90 (Ni MPs), 106 (Ni20 NPs), and 223 (NiO MPs). The NPs of NiO did not cause any cytotoxic effect at any of the time points tested. At 72 h, no or a minor number of cells were viable on doses above 5 μg Ni/mL for NiSO_4_·6H_2_O and Ni_3_S_2_, i.e., the soluble salt and the Ni subsulfide substance showing the highest biodissolution (62% of the transferred mass of Ni) compared to the other Ni substances (<15%) as described above.

**Figure 4:**
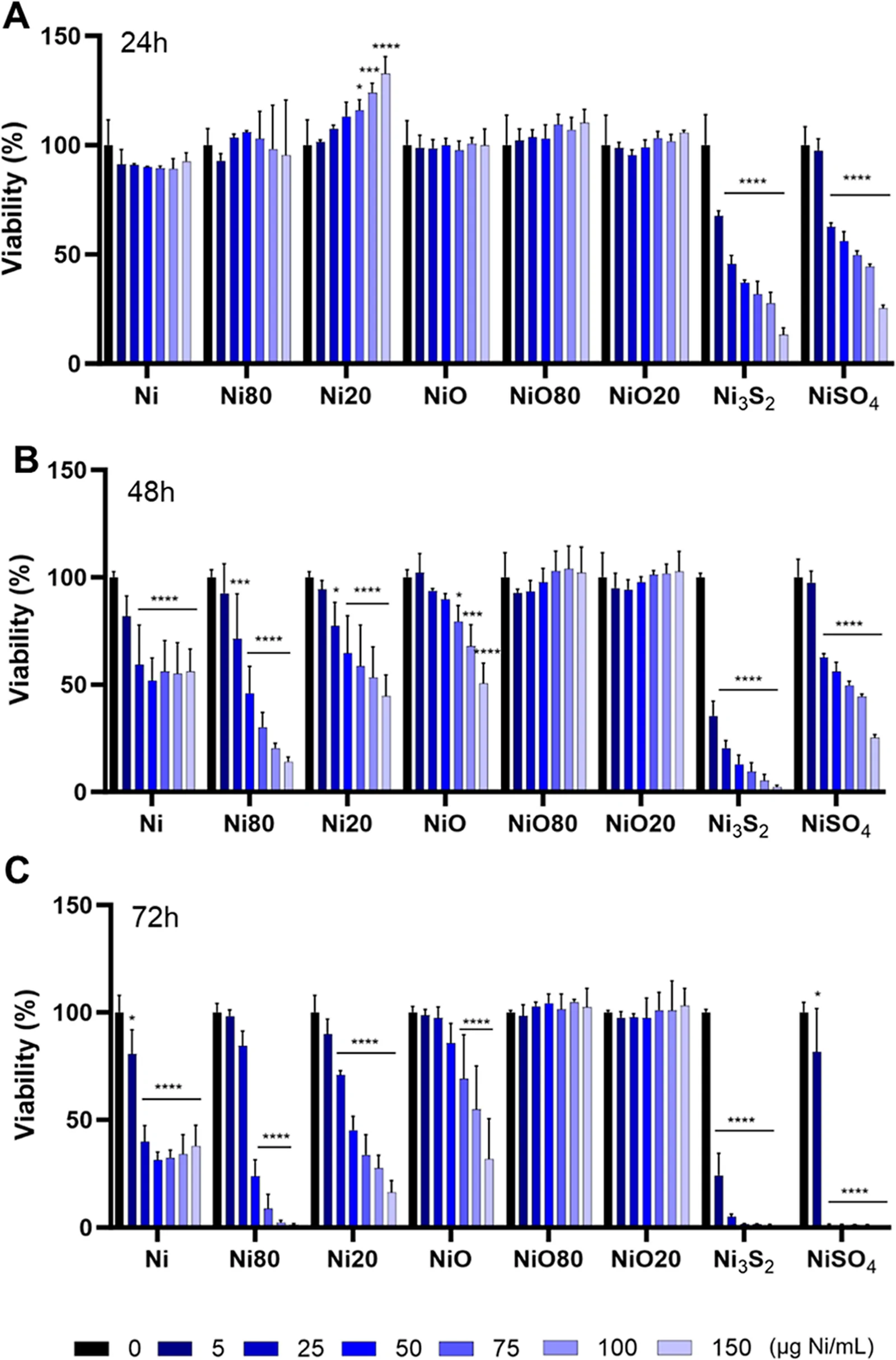
Cytotoxicity of all Ni substances in BEAS-2B cells after exposure for 24 h (A), 48 h (B), or 72 h (C). The cells were exposed to 5–150 μg Ni/mL. The bars show the mean + SD (*n* = 3). Significant difference compared to controls are marked with asterisks (*p < 0.05, **p < 0.01, ***p < 0.001, ****p < 0.0001).

The dose–response curve at 48 h is also presented as a scatter plot in the Supporting Information (Figure S1). When the dose is expressed as substance mass per mL (instead of added Ni mass per mL), Ni_3_S_2_ remains the most toxic substance, with an EC50 of 14 μg/mL at this time point. At this dose-metric, the water-soluble salt, NiSO_4_·6H_2_O, exhibits a substantially higher EC50 of 283 μg/mL, indicating lower toxicity (Figure S1). When adjusting the dose taking into account the mass transfer (adjusted dose, based on separate experiments), it was even more obvious that Ni_3_S_2_ was by far the most cytotoxic substance (Figure S1). When comparing biodissolution with cytotoxicity at a nominal dose of 100 μg Ni/mL, it was evident that only the most soluble substances induced cytotoxic effects after 24 h. At later time points, even substances with minimal Ni release such as Ni metal MPs, which released only 0.3–0.5%, exhibited toxic effects (see Figure S2). There was a significant correlation between cytotoxicity at 100 μg/mL and Ni release at 72 h (Spearman’s rank correlation, *r* = 0.8, *p* = 0.02), whereas no such relationship was observed at 24 or 48 h.

### TEM Imaging of the Cellular Uptake

The cell uptake of all Ni substances was explored using TEM imaging. The results showed that all substances, except for the water-soluble salt (NiSO_4_·6H_2_O), were clearly visible in the cells as particles. The particles were observed as aggregates/agglomerates in membrane-bound structures in the cytosol. No particles were observed in the membrane-bound structures in the case of NiSO_4_·6H_2_O since the salt was completely dissolved and hence primarily taken up as Ni^2+^ ions (or possibly as weak complexes). The particle uptake is most likely associated with the internalization of the smaller-sized particles bound to the micron-sized particles in the case of Ni and NiO MPs and Ni_3_S_2_, as well as nanoscale particles within the micron-sized aggregates of Ni80, Ni20, NiO80, and NiO20 (see Figure 1).

### Time and Dose-Dependent Uptake Following Exposure to the Various Ni Substances

Next, we explored the amount of Ni taken up into the cells quantitatively using GF-AAS. However, the methodology cannot differentiate between internalized Ni and Ni adhering to the cell surface, which possibly remained after washing. Therefore, we cannot ignore the fact that the reported uptake values may overestimate the internalized Ni. First, we investigated this at all three time points, i.e., 24 h, 48 h, and 72 h. The results showed a rather high variation among the replicates for each substance, but it was obvious that the cell uptake was rather similar across time points among the various Ni substances but much lower for the soluble Ni salt (NiSO_4_·6H_2_O), Figure S3. Since the first set of data showed rather high variation, we made some method changes (mainly included an extra wash) and explored even a shorter time point (4 h) as well as two doses (5 and 10 μg Ni/mL). The most striking result was the low uptake of soluble Ni (NiSO_4_·6H_2_O) compared to the less soluble Ni substances, Figure 6. As expected, the uptake was clearly higher in the higher dose. Ni metal showed the highest apparent uptake of all Ni substances, being significantly higher (ANOVA, adj *p*-value <0.05) compared to NiO20, NiO80, Ni_3_S_2_ (as well as the soluble Ni). The uptake at 24 h was not higher than at 4 h, indicating a lack of time-dependent increase in the apparent cell uptake and a potentially rapid interaction of the Ni substances with the cells.

**Figure 5:**
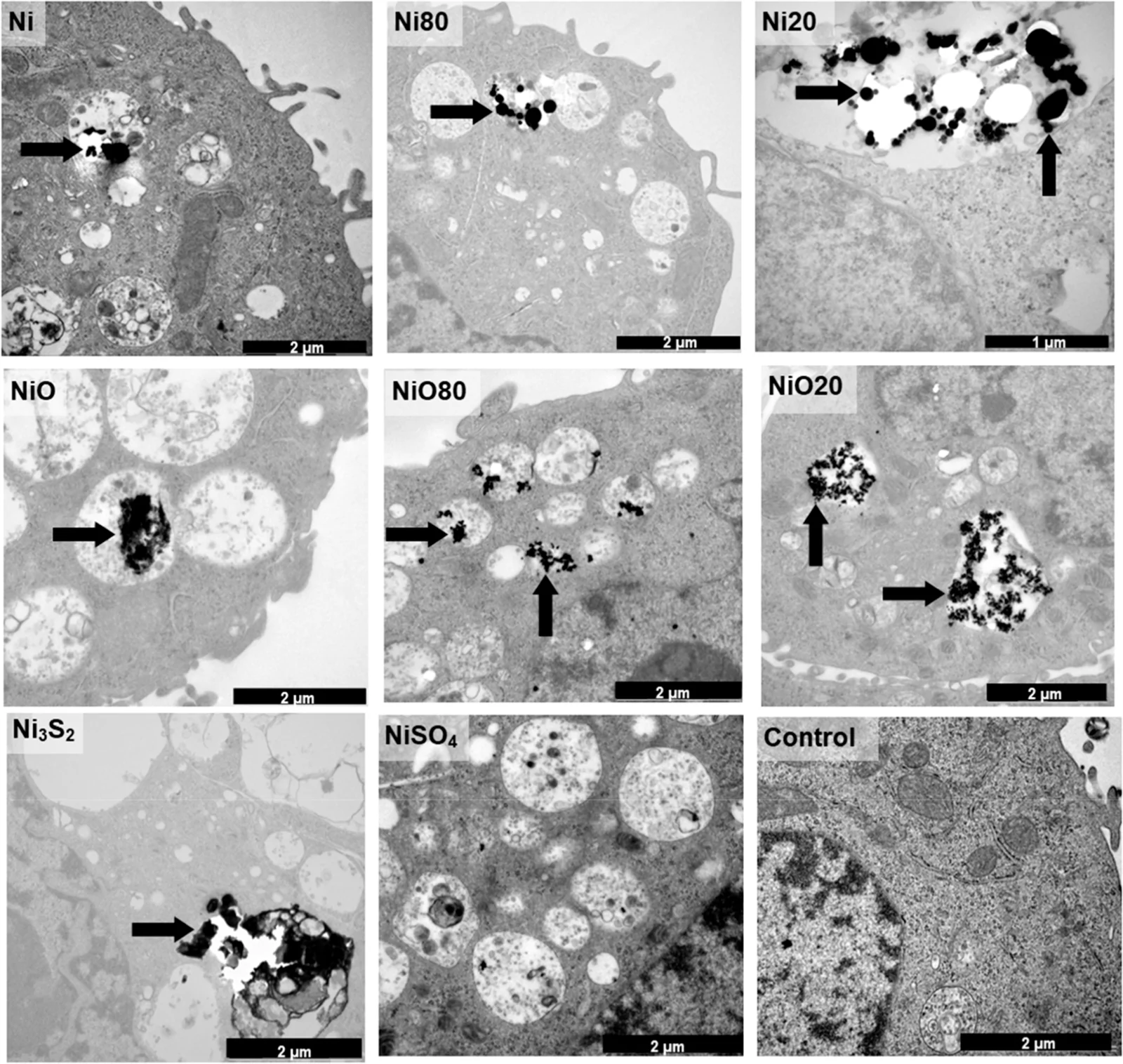
TEM images showing uptake of all Ni substances, except NiSO_4_·6H_2_O, in BEAS-2B cells following 48 h exposure. Arrows indicate Ni substances clearly observed in vacuoles of the cells.

**Figure 6:**
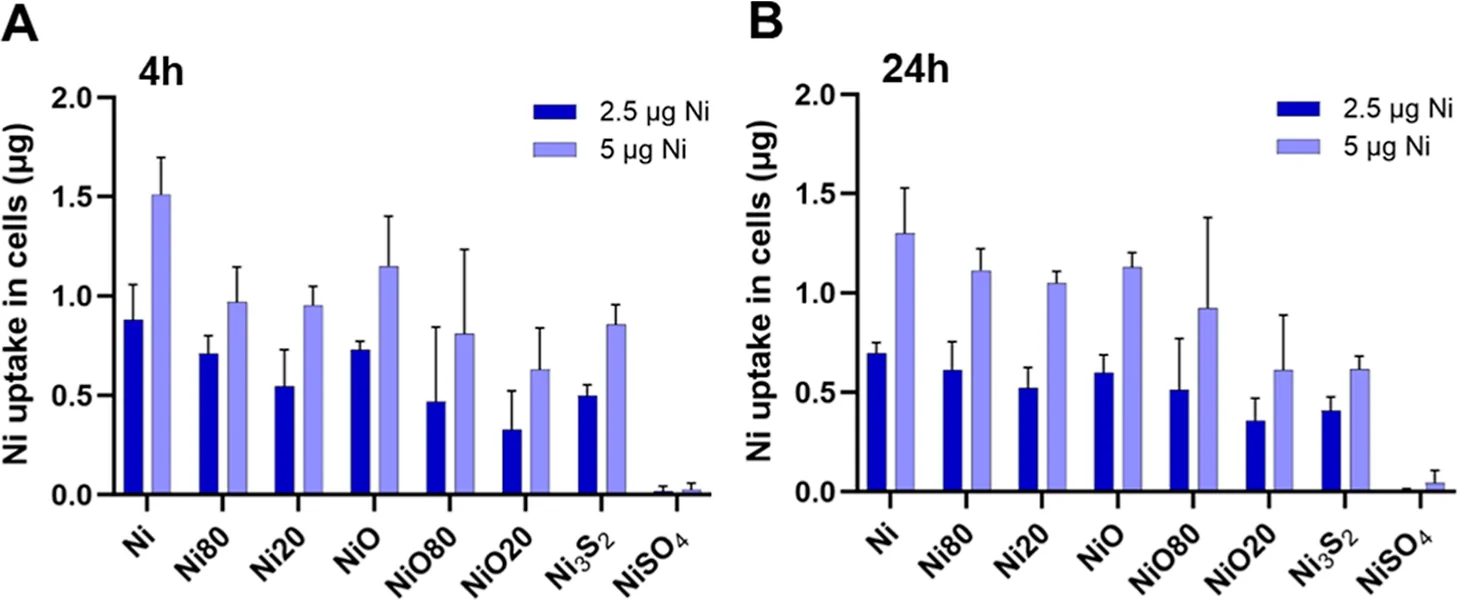
Uptake of the various Ni substances in BEAS-2B cells after 4 h (A) or 24 h (B) exposure. The uptake can also include Ni firmly attached to the cells or in the cell membrane. Cells were exposed to 2.5 and 5 μg Ni (5 and 10 μg Ni/mL) nominal doses. The bars show the mean + SD (*n* = 3).

Ni levels in the wash fraction were also analyzed. As shown in Figure S4A, nearly all Ni of soluble Ni (NiSO_4_·6H_2_O) was recovered in this fraction. Using these data, we calculated the percentage of Ni associated with the cell fraction relative to the total Ni recovered (cell + wash). The results indicated that for most sparingly soluble Ni substances, approximately 50–70% of the added Ni was associated with the cells (Figure S4B).

We next performed recovery experiments and investigated whether the uptake would decrease after removing additional Ni exposure. Thus, cells were exposed for 4 h, washed, and then fresh media added, followed by a recovery time of 20 h. The results showed that almost no soluble Ni (from NiSO_4_·6H_2_O) could be detected in the cells after recovery (although starting from an already very low uptake level) and a slightly lower uptake (15–30%) was observed for several of the other Ni substances (Ni, Ni20, Ni80, and NiO). The variation between the samples was, however, too large to be statistically significant, Figure 7. Ni_3_S_2_ showed less variation and with no reduction observed after the recovery period.

**Figure 7:**
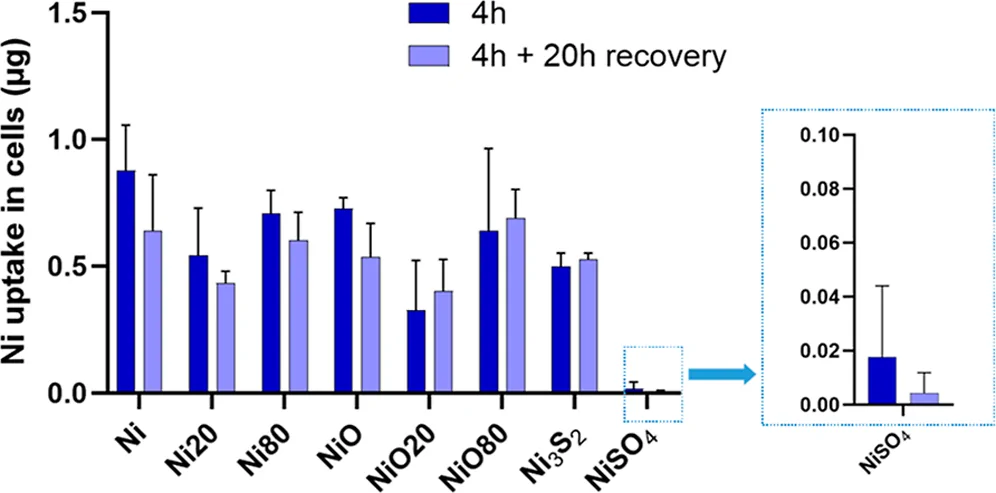
Uptake of the various Ni substances in BEAS-2B cells after 4 h compared to 4 h followed by 20 h recovery. Cells were exposed to 2.5 μg Ni (5 μg/mL). The bars show the mean + SD (*n* = 3).

### Nonparticulate (“Dissolved”) Ni in Cytosol and Nucleus Following Exposure to the Various Ni Substances

Next, we performed fractionation of the cells into a cytosolic, nuclear, and particulate fraction, respectively, and measured the extent of Ni in the cytosolic and nuclear fractions. During the fractionation procedure, particles are removed through centrifugation in the various steps. Here, the measured intracellular Ni (in cytosol or nucleus) is considered to reflect the amount of Ni released within the cells from the sparingly soluble Ni substances. The results showed that the cytosolic level was the lowest for NiSO_4_·6H_2_O (0.03 pg/cell = 30 ng/10^6^ cells) and highest for Ni MPs and Ni20 (approximately 4 pg/cell = 4000 ng/10^6^ cells), see Figure 8A. The same trend was observed for the nuclear fraction with the lowest levels for soluble Ni (0.0002 pg/cell = 0.2 ng/10^6^ cells) and highest for Ni20 (1.2 pg/cell = 1200 ng 10^6^ cells), followed by Ni MPs (0.6 pg/cell = 600 ng 10^6^ cells) and Ni_3_S_2_ (0.2 pg/cell = 200 ng 10^6^ cells), see Figure 8B. The nuclear to cytoplasmic ratio was highest for Ni20 and Ni_3_S_2_ (∼1:5) and lowest for Ni80, NiO80, and NiSO_4_ (between 1:10 and 1:100).

**Figure 8:**
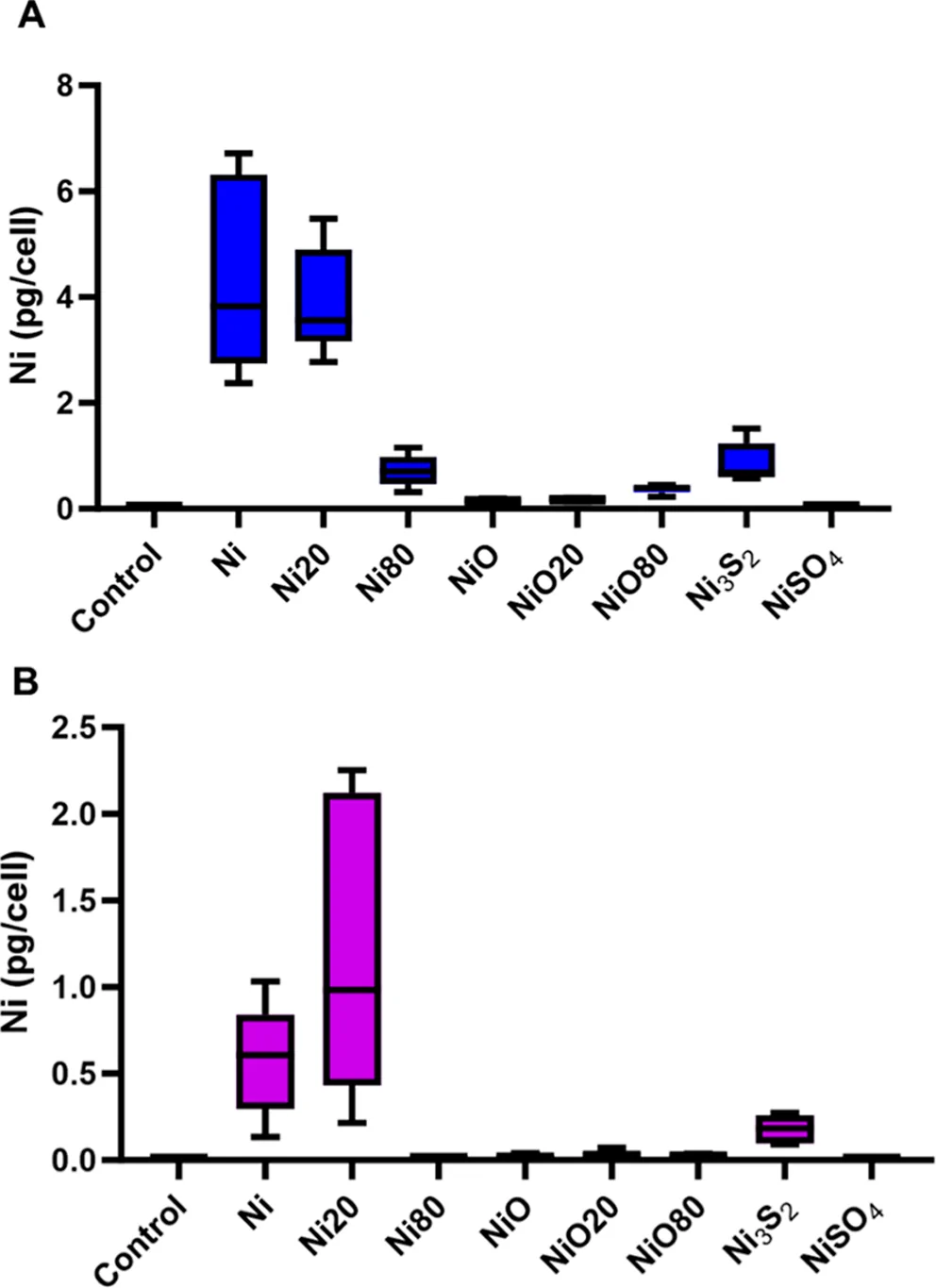
Ni in the cytosolic and nuclear fraction. A fractionation procedure was used to enable analysis of nonparticulate (“dissolved”) Ni in the cytosol (A) and nucleus (B) following BEAS-2B exposure to 5 μg Ni/mL the various Ni substances for 48 h. The box shows the 25th to 75th percentiles, whiskers shows the min and max values, and the line indicates median (*n* = 5).

### Ni Localization Using Super-Resolution Microscopy

To explore the possibility to visualize Ni dissolved from the Ni substances within the cells, super-resolution microscopy was used with the Newport Green staining that binds to Ni^2+^ among other ions. Figure 9 shows the cell morphology, cytoskeletal structure (Phalloidin-red-quadrant 1), the nucleus (Hoechst-blue-quadrant 2), presence of intracellular Ni^2+^ ions (Newport Green-green-quadrant 3), and overlay image (quadrant 4). A weak signal of Newport Green was observed in both the cytoplasmic and nuclear compartments of the untreated control, Figure 9A. For the Ni MPs as well as Ni_3_S_2_, a slight increase of Newport Green intensity was seen in the cytoplasmic compartment and possibly also in the nuclear compartment (although less clear) (Figure 9B,C). For all other Ni substances, no differences in the signal compared to control were observed (see Figure S5). Taken together, it was difficult to discern an overall significant increase in the Newport Green signal, indicative of Ni ions or Ni labile complexes, in the nuclear compartment for the various exposures.

**Figure 9:**
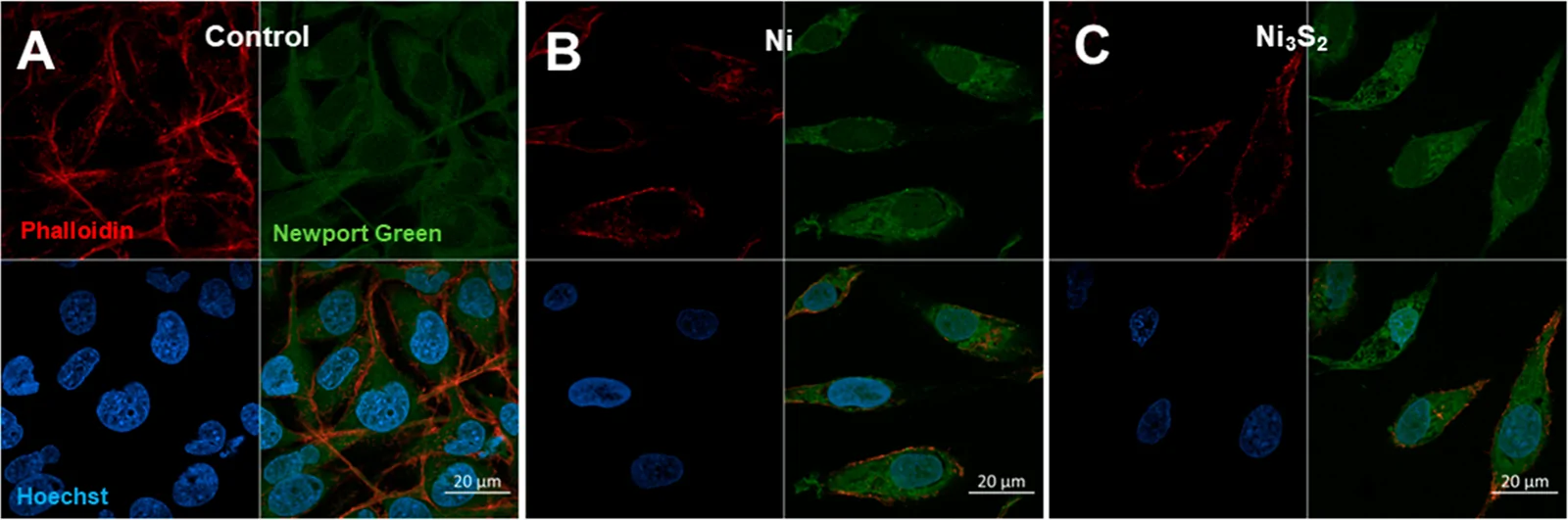
Newport Green distribution in BEAS-2B cells exposed for 24 h to the various Ni substances. Each image presents the cell morphology and cytoskeletal structure in quadrant 1 (Phalloidin-red), the nucleus in quadrant 2 (Hoechst-blue), the presence of intracellular Ni^2+^ ions in quadrant 3 (Newport Green–green), and the overlay image in quadrant 4. The cells were exposed for 24 h to 50 μg Ni/mL. The figure shows images from untreated controls (A), and after exposure to Ni MPs (B) and Ni_3_S_2_ (C). The scale bars indicate 20 μm.

## Discussion

The impact of physicochemical properties, such as particle size and biodissolution, on the cellular uptake and intracellular distribution of Ni substances remains insufficiently characterized. To address this knowledge gap, the present study systematically investigated nano- and micrometer-sized powder particles of eight Ni substances with different properties. We employed a combination of qualitative and quantitative analyses to assess the total cellular uptake and the distribution of dissolved Ni^2+^ from the substances within the cytosolic and nuclear fractions. We selected the mass of Ni as the dose metric because it provides the most robust and experimentally verifiable basis for comparing the different Ni-containing materials investigated in this study. While particle number and surface area are recognized as important dose metrics in nanotoxicology, both can be substantially altered by agglomeration under cell-culture conditions.

### Cellular Uptake and Intracellular Distribution

Our data clearly demonstrates minimal cellular uptake of soluble Ni (from completely dissolved NiSO_4_·6H_2_O) in BEAS-2B cells (Figure 6), with levels further declining upon removal of the Ni ion source in recovery experiments (Figure 7). This is in line with previous studies such as Edwards et al.^29^ showing similar uptake (0.02 pg/cell vs 0.03 pg/cell in our study) following exposure of macrophages to 10 μg/mL Ni ions (from NiSO_4_·6H_2_O). Various ligands can affect the cell uptake and Abbracchio et al.^30^ reported, for example, that the Ni ion uptake by cell lines was reduced by approximately 14-fold when the culture medium contained physiological levels of amino acids and proteins, compared to a minimal medium consisting only of glucose and salts. Clearly, low levels of Ni ions will be present in the cell culture medium due to complexation.^22^ In contrast, the carcinogenic Ni_3_S_2_ showed substantial apparent cellular uptake, with no reduction in intracellular levels 20 h after removal (Figure 7). This pattern of high uptake, also visualized using TEM imaging (Figure 5), and limited clearance during recovery, was also observed for the other poorly soluble Ni substances. It is important to note that the GF-AAS method used here does not distinguish between Ni in the cell and Ni bound to the extracellular cell membrane (remaining after washing). The measured cellular Ni is likely made up of Ni taken up into the cell and Ni bound to the cell surface, with possible differences in the extracellular Ni binding of the different Ni substances contributing to the apparent cell uptake. Cellular adherence can be strongly influenced by the biocorona formed on metal particles in physiological media.^31,32^ As the biocorona largely masks the original particle surface, cell–particle interactions are primarily determined by corona composition and properties, including receptor engagement and membrane adhesion. Accordingly, even negatively charged biocorona-coated particles may interact with negatively charged cell membranes through protein–receptor binding, electrostatic heterogeneity within the corona, or cation-mediated bridging.^31,32^ Previous studies have investigated the reversibility of Ni taken up into different cell types and the results show time and other factors as drivers of uptake and reversibility or retention.^29,33,34^

An important aim of our study was to determine whether any Ni was released intracellularly and localized within the nucleus. Quantitative analysis using GF-AAS indicated some dissolved Ni in the cytosolic and nuclear fractions for all Ni substances, but more pronounced for Ni metal MPs, Ni20, and Ni_3_S_2_. For Ni metal and Ni_3_S_2_, the data were further supported by the super-resolution microscopy-based qualitative analysis, although clear conclusions were difficult to draw from these images. The Newport Green staining in our study was not as intense and different between control and Ni-exposed cells as in the study by Ke et al.,^35^ which showed that both NiCl_2_ and NiS were taken up by human lung carcinoma A549 cells, resulting in Ni ion accumulation in the cytoplasm and nucleus. Furthermore, when the exposure to the Ni substance ceased, Ni ions persisted in the NiS-exposed cells longer than in the NiCl_2_-exposed ones.^35^ Pietruska et al.^36^ also used the fluorescent probe Newport Green to demonstrate intracellular Ni release within 24 h of exposure to NiCl_2_ or NiO NPs and within 48 h following exposure to metallic Ni NPs. Interestingly, the NiO NPs in their study released nearly 50% of the available Ni within 24 h, which is substantially higher than the release observed for the NiO NPs in the present study (approximately 3%), a result consistent with our previous findings.^22^

The nuclear extract kit used in the present study was previously employed by Strauch et al.^37^ to assess the cellular uptake and nuclear localization of copper (Cu) substances. Similarly, Fletcher et al.^38^ used a comparable fractionation approach to investigate the intracellular distribution of Ni substances in AS52 cells, a cell line derived from CHO cells. In their study, varying doses were selected based on differences in cytotoxicity. They reported cytosolic Ni levels of 35–250 ng per 10^6^ cells (excluding NiCO_3_) and nuclear levels of ∼6–80 ng per 10^6^ cells. In our study, the cytosolic fraction ranged from 30 to 4000 ng per 10^6^ cells, while the nuclear fraction ranged from 0.2 to 1200 ng per 10^6^ cells. The nuclear-to-cytoplasmic ratio was relatively comparable to our findings. One similarity between the studies was the high cytotoxicity of Ni_3_S_2_ and combined with an ability to efficiently deliver Ni ions into cells.

### Biodissolution, Surface Area, and Cytotoxicity

Ni MP and Ni20 showed highest apparent content of nuclear Ni. This is particularly relevant considering our findings in Latvala et al.,^22^ which demonstrated high dissolution of Ni NPs and Ni MPs in artificial lysosomal fluid (ALF). Possibly, the dissolution in ALF better correlates with Ni in the cytosolic and nuclear fraction. Since lysosomes containing particles can occasionally localize near the nucleus, this may facilitate enhanced nuclear uptake of Ni^2+^ ions, especially compared to the minimal uptake observed for extracellular Ni ions or substances with limited lysosomal solubility.^13,19,20^ Notably, our findings in Latvala et al.^22^ showed that the dissolution of Ni NPs and MPs in ALF was substantially higher, up to 100%, compared to only 1–3% in cell culture media. The dissolution characteristics of Ni NPs and MPs depend to a significant extent on the surface characteristics. Our study showed a coexistence of NiO and Ni­(OH)_2_ on Ni metal, which is consistent with previous studies that indicated that NiO typically forms a compact, inner oxide layer, whereas Ni­(OH)_2_ develops as a hydrated outer layer.^39^ The latter possesses a brucite-like, layered double hydroxide structure characterized by weak interlayer bonding, resulting in higher solubility compared to the dense cubic structure of NiO.

Similar findings have been observed for NiO and Ni­(OH)_2_ NPs (15–20 nm sized particles) showing Ni­(OH)_2_ to be more soluble compared to NiO at cytosolic and lysosomal pH conditions.^40^ For the NiO particles, the released amount of Ni was the highest from the NiO MPs (9%) compared with the NiO80 and NiO20 NPs (≤3%). This discrepancy cannot be explained by differences in the surface composition but possibly be a result of differences in exposed surface areas judged from the DLS measurements. However, this is only speculative as the actual surface area exposed to solution depends on the packing density and porosity of the aggregates, a measure not possible to determine from the data, although the specific surface area (as determined by BET) previously reported, suggest highest area for NiO MP (96 m^2^/g) compared to NiO80 NPs (50 m^2^/g) and NiO20 NPs (74 m^2^/g).^21^ Similar biodissolution trends as our study were reported by Lyons-Darden et al.^21^ in simulated interstitial fluid (pH 7.4), showing lower Ni release from Ni MPs compared with Ni NPs, higher release from Ni80 NPs compared with Ni20 NPs, and slightly greater Ni release from NiO MPs than from NiO NPs, with overall very similar masses of dissolved Ni observed.^21^ The BET area was lower for the Ni metal particles compared to the NiO (Ni MP: 2.6 m^2^/g; Ni80 NPs: 8 m^2^/g, Ni20 NPs: 27 m^2^/g) and observed differences in biodissolution could not be related to differences in surface area measured under dry conditions.^21^ The underlying explanation for the low dissolution of the Ni MPs is most probably related to their strong tendency to aggregate and the high polydispersity in solution, see Table 1, resulting in rapid sedimentation, also visually observed during the experiments. The observed trend is consistent with the XPS results with the surface oxide thickness increasing from the Ni80 NPs to Ni MPs.

The NiO NPs showed low biodissolution and no cytotoxicity, despite their cellular uptake. NiO has, in previous studies, shown a variation in toxicity, which partly can be explained by different production processes (e.g., the calcining temperature) which affect their biodissolution and biological effects.^41^ For example, an inhalation study in rats suggests that NiO (green) is a weakly positive carcinogen in rats compared to the more potent Ni_3_S_2_.^41^ Overall, the limited cytotoxicity of NiO and high cytotoxicity of Ni_3_S_2_ and NiSO_4_·6H_2_O, as noted in our study, is in line with acute and subacute inhalation studies in rats.^42,43^

### Study Limitations

It should be emphasized that the present study has several limitations. First, the AAS measurements quantify total cell-associated Ni and do not distinguish between intracellularly localized and membrane-bound Ni. More advanced wash procedures, including, e.g., wash steps with EDTA, citrate, and cysteine,^44^ or more advanced quantitative imaging, could help discriminate between these fractions in future studies. Second, Newport Green is not specific for Ni ions and labile complexes as it also responds to other divalent transition metals, including, e.g., Zn, Cu, and Fe.^45^ Consequently, interference from endogenous cellular metals could not be completely excluded. Third, our data indicate pronounced agglomeration and rapid sedimentation of all sparingly soluble Ni substances. As particle agglomeration influences sedimentation, transport, and the effective dose delivered to cells, differences in cellular dosimetry may have contributed to the observed uptake patterns.^46^ Although in vitro dosimetry modeling could have provided a more accurate estimate of the delivered dose, such analyses were beyond the scope of the present study. Finally, while the conditions used in this study allow for comparison of cellular uptake and intracellular distribution across different Ni substances, they do not fully recapitulate the complexity of the lung microenvironment in vivo. Following inhalation exposure, particle behavior and cellular interactions may be influenced by factors such as mucus, pulmonary surfactant, immune cells, and clearance mechanisms (including the rapid clearance of soluble Ni). In addition, inflammatory responses may modify metal dissolution, cellular uptake, and redistribution. Consequently, the present results should be interpreted as mechanistic insights obtained in a simplified epithelial cell model rather than as a direct prediction of in vivo lung responses.

## Conclusions

To facilitate a comparison of the data, the main findings are summarized in Table 2. Several trends were apparent. Most notably, all NiO particles tested (both MPs and NPs) exhibited lower cytotoxicity than did Ni metal particles and Ni_3_S_2_. This difference cannot be readily explained by the lower cellular uptake or lower dissolution in the cell culture medium and may instead be related to differences in intracellular dissolution and bioavailability. However, some findings cannot be readily explained. For example, Ni metal particles (MPs and NPs) showed somewhat higher total, cytosolic, and nuclear nickel uptake than Ni_3_S_2_ yet induced markedly lower cytotoxicity. This suggests that factors other than the total cellular nickel burden contribute to the observed toxic effects. In conclusion, this study highlights the complex interplay among biodissolution, cellular uptake, and cytotoxicity of Ni substances.

**Table 2:** Overview of the Main Findings of the Study^a^

substance	Ni release in cell media (48 h, % Ni)	Ni uptake TEM and AAS (total μg^b^ at 24 h)	Ni in cytosol (48 h, pg/cell)	Ni in nucleus (48 h, pg/cell)	EC50 (48 h, μg Ni/mL)
Ni	0.3	yes (0.69)	4.39	0.576	90
Ni20	2.9	yes (0.52)	3.94	1.218	106
Ni80	9.0	yes (0.61)	0.72	0.007	39
NiO	4.4	yes (0.60)	0.15	0.017	223
NiO20	2.3	yes (0.36)	0.17	0.033	-^c^
NiO80	2.9	yes (0.51)	0.37	0.021	-^c^
Ni_3_S_2_	55.1	yes (0.41)	0.87	0.179	3.5
NiSO_4_	∼100	little (0.01)	0.03	0.002	63

a The table summarizes data shown in Figure 3 (biodissolution), Figures 5 and 6 (uptake), Figure 8 (Ni in cytosol and nucleus), as well as Figure 4 (cytotoxicity).

b Total μg Ni in cells after exposure to 2.5 μg Ni (5 μg Ni/mL).

c No cytotoxicity was observed.

## Supplementary Material



## Notes

Dose–response of cytotoxicity when considering the nominal dose as μg Ni/mL, the nominal dose as μg substance/mL and the μg Ni/mL calculated added dose; correlation between biodissolution (Ni release) in cell media and cytotoxicity at 100 μg/mL; uptake of various Ni substances at 24 h, 48 h, and 72 h; uptake of various Ni substances in BEAS-2B cells after 24 h; and Newport Green distribution in BEAS-2B cells exposed for 24 h to the various Ni substances in concentrations of 50 μg Ni/mL (PDF)

## References

[1] Geological Survey, U. S. Mineral Commodity Summaries 2024: Nickel; U.S. Geological Survey: Reston, VA, 2024. https://pubs.usgs.gov/periodicals/mcs2024/mcs2024-nickel.pdf.

[2] Buxton S., Garman E., Heim K. E., Lyons-Darden T., Schlekat C. E., Taylor M. D., Oller A. R. (2019). Concise
Review of
Nickel Human Health Toxicology and Ecotoxicology. Inorganics.

[3] International Agency for Research on Cancer (IARC) . IARC Monographs on the Evaluation of Carcinogenic Risks to Humans, Vol. 49: Chromium, Nickel and Welding; IARC: Lyon, France, 1990. PMC7681426 2232124

[4] International Agency for Research on Cancer (IARC) . IARC Monographs on the Evaluation of Carcinogenic Risks to Humans, Vol. 100C: Arsenic, Metals, Fibres, and Dusts; IARC: Lyon, France, 2012. PMC4781271 23189751

[5] Muñoz A., Costa M. (2012). Elucidating the mechanisms of nickel
compound uptake: a review of
particulate and nano-nickel endocytosis and toxicity. Toxicol. Appl. Pharmacol..

[6] Li W., Zhou J., Boon D., Fan T., Anneser E., Goodman J. E., Prueitt R. L. (2024). Nickel in ambient particulate matter
and respiratory or cardiovascular outcomes: A critical review. Environ. Pollut..

[7] Henderson R. G., Cappellini D., Seilkop S. K., Bates H. K., Oller A. R. (2012). Oral bioaccessibility
testing and read-across hazard assessment of nickel compounds. Regul. Toxicol. Pharmacol..

[8] National Toxicology Program . NTP Technical Report on the Toxicology and Carcinogenesis Studies of Nickel Oxide (CAS No. 1313–99–1) in F344/N Rats and B6C3F1Mice (Inhalation Studies), Technical Report Series No. 451; U.S. Department of Health and Human Services, Public Health Service, National Institutes of Health: Research Triangle Park, NC, 1996. 12594524

[9] National Toxicology Program NTP Toxicology and Carcinogenesis Studies of Nickel Sulfate Hexahydrate (CAS No. 10101–97–0) in F344 Rats and B6C3F1Mice (Inhalation Studies), Technical Report Series No. 454; U.S. Department of Health and Human Services, Public Health Service, National Institutes of Health: Research Triangle Park, NC, 1996. 12587012

[10] National Toxicology Program NTP Technical Report on the Toxicology and Carcinogenesis Studies of Nickel Subsulfide (CAS No. 12035–72–2) in F344/N Rats and B6C3F1Mice (Inhalation Studies), Technical Report Series No. 453; U.S. Department of Health and Human Services, Public Health Service, National Institutes of Health: Research Triangle Park, NC, 1996. 12594522

[11] Heim K. E., Bates H. K., Rush R. E., Oller A. R. (2007). Oral carcinogenicity
study with nickel sulfate hexahydrate in Fischer 344 rats. Toxicol. Appl. Pharmacol..

[12] Oller A. R., Kirkpatrick D. T., Radovsky A., Bates H. K. (2008). Inhalation
carcinogenicity
study with nickel metal powder in Wistar rats. Toxicol. Appl. Pharmacol..

[13] Costa M., Mollenhauer H. H. (1980). Carcinogenic activity of particulate
nickel compounds
is proportional to their cellular uptake. Science.

[14] Costa M., Simmons-Hansen J., Bedrossian C. W., Bonura J., Caprioli R. M. (1981). Phagocytosis,
cellular distribution, and carcinogenic activity of particulate nickel
compounds in tissue culture. Cancer Res..

[15] Skoglund S., Hedberg J., Yunda E., Godymchuk A., Blomberg E., Odnevall Wallinder I. (2017). Difficulties
and flaws in performing
accurate determinations of zeta potentials of metal nanoparticles
in complex solutions-Four case studies. PLoS
One.

[16] Davidson T., Chen H., Garrick M. D., D’Angelo G., Costa M. (2005). Soluble nickel interferes with cellular iron homeostasis. Mol. Cell. Biochem..

[17] Refsvik T., Andreassen T. (1995). Surface binding and uptake of nickel­(II)
in human epithelial
kidney cells: modulation by ionomycin, nicardipine and metals. Carcinogenesis.

[18] Blomberg E., Wang X., Herting G., Khort A., Arora A., Buxton S., Lyons-Darden T., Karlsson H. L., Odnevall I. (2025). Effects of
sonication on particle dispersions from a size, biodissolution, cytotoxicity
and transferred dose perspective - a case study on nickel and nickel
oxide particles. PLoS One.

[19] Costa M., Mollenhauer H. H. (1980). Phagocytosis
of nickel subsulfide particles during
the early stages of neoplastic transformation in tissue culture. Cancer Res..

[20] Goodman J. E., Prueitt R. L., Thakali S., Oller A. R. (2011). The nickel ion bioavailability
model of the carcinogenic potential of nickel-containing substances
in the lung. Crit. Rev. Toxicol..

[21] Lyons-Darden T., Heim K. E., Han L., Haines L., Sayes C. M., Oller A. R. (2024). Bioaccessibility
of Metallic Nickel and Nickel Oxide
Nanoparticles in Four Simulated Biological Fluids. Nanomaterials.

[22] Latvala S., Hedberg J., Di Bucchianico S., Möller L., Odnevall Wallinder I., Elihn K., Karlsson H. L. (2016). Nickel
Release,
ROS Generation and Toxicity of Ni and NiO Micro- and Nanoparticles. PLoS One.

[23] Di
Bucchianico S., Gliga A. R., Akerlund E., Skoglund S., Wallinder I. O., Fadeel B., Karlsson H. L. (2018). Calcium-dependent
cyto- and genotoxicity of nickel metal and nickel oxide nanoparticles
in human lung cells. Part. Fibre Toxicol..

[24] Gliga A. R., Di Bucchianico S., Akerlund E., Karlsson H. L. (2020). Transcriptome Profiling
and Toxicity Following Long-Term, Low Dose Exposure of Human Lung
Cells to Ni and NiO Nanoparticles-Comparison with NiCl(2). Nanomaterials.

[25] Grosvenor A. P., Biesinger M. C., Smart R. S. C., McIntyre N. S. (2006). New interpretations
of XPS spectra of nickel metal and oxides. Surf.
Sci..

[26] Biesinger M. C., Payne B. P., Grosvenor A. P., Lau L. W. M., Gerson A. R., Smart R. S. C. (2011). Resolving surface chemical states in XPS analysis of
first row transition metals, oxides and hydroxides: Cr, Mn, Fe, Co
and Ni. Appl. Surf. Sci..

[27] Kessler A., Hedberg J., McCarrick S., Karlsson H. L., Blomberg E., Odnevall I. (2021). Adsorption of Horseradish
Peroxidase on Metallic Nanoparticles:
Effects on Reactive Oxygen Species Detection Using 2’,7’-Dichlorofluorescin
Diacetate. Chem. Res. Toxicol..

[28] Pradhan S., Hedberg J., Blomberg E., Wold S., Odnevall
Wallinder I. (2016). Effect of sonication on particle dispersion, administered
dose and metal release of non-functionalized, non-inert metal nanoparticles. J. Nanopart. Res..

[29] Edwards D. L., Wataha J. C., Hanks C. T. (1998). Uptake
and reversibility of uptake
of nickel by human macrophages. J. Oral Rehabil..

[30] Abbracchio M. P., Evans R. M., Heck J. D., Cantoni O., Costa M. (1982). The regulation
of ionic nickel uptake and cytotoxicity by specific amino acids and
serum components. Biol. Trace Elem. Res..

[31] Monopoli M. P., Åberg C., Salvati A., Dawson K. A. (2012). Biomolecular coronas
provide the biological identity of nanosized materials. Nat. Nanotechnol..

[32] Aliyandi A., Zuhorn I. S., Salvati A. (2020). Disentangling Biomolecular Corona
Interactions With Cell Receptors and Implications for Targeting of
Nanomedicines. Front. Bioeng. Biotechnol..

[33] Wataha J. C., Hanks C. T., Craig R. G. (1993). Uptake of metal
cations by fibroblasts
in vitro. J. Biomed Mater. Res..

[34] Wataha J. C., Hanks C. T., Craig R. G. (1994). In vitro
effects of metal ions on
cellular metabolism and the correlation between these effects and
the uptake of the ions. J. Biomed Mater. Res..

[35] Ke Q., Davidson T., Kluz T., Oller A., Costa M. (2007). Fluorescent
tracking of nickel ions in human cultured cells. Toxicol. Appl. Pharmacol..

[36] Pietruska J. R., Liu X., Smith A., McNeil K., Weston P., Zhitkovich A., Hurt R., Kane A. B. (2011). Bioavailability,
Intracellular Mobilization
of Nickel, and HIF-1α Activation in Human Lung Epithelial Cells
Exposed to Metallic Nickel and Nickel Oxide Nanoparticles. Toxicol. Sci..

[37] Strauch B. M., Niemand R. K., Winkelbeiner N. L., Hartwig A. (2017). Comparison between
micro- and nanosized copper oxide and water soluble copper chloride:
interrelationship between intracellular copper concentrations, oxidative
stress and DNA damage response in human lung cells. Part. Fibre Toxicol..

[38] Fletcher G. G., Rossetto F. E., Turnbull J. D., Nieboer E. (1994). Toxicity, uptake, and
mutagenicity of particulate and soluble nickel compounds. Environ. Health Perspect..

[39] Graedel T. E., Leygraf C. (2000). Corrosion Mechanisms for Nickel Exposed
to the Atmosphere. J. Electrochem. Soc..

[40] Cambre M. H., Holl N. J., Wang B., Harper L., Lee H. J., Chusuei C. C., Hou F. Y. S., Williams E. T., Argo J. D., Pandey R. R., Huang Y. W. (2020). Cytotoxicity of NiO and Ni­(OH)(2)
Nanoparticles Is Mediated by Oxidative Stress-Induced Cell Death and
Suppression of Cell Proliferation. Int. J. Mol.
Sci..

[41] Oller A. R., Costa M., Oberdörster G. (1997). Carcinogenicity
assessment of selected
nickel compounds. Toxicol. Appl. Pharmacol..

[42] Lyons-Darden T., Blum J. L., Schooley M. W., Ellis M., Durando J., Merrill D., Oller A. R. (2023). An Assessment
of the Oral and Inhalation
Acute Toxicity of Nickel Oxide Nanoparticles in Rats. Nanomaterials.

[43] Oller A. R., Buxton S., March T. H., Benson J. M. (2023). Comparative pulmonary
and genotoxic responses to inhaled nickel subsulfide and nickel sulfate
in F344 rats. J. Appl. Toxicol..

[44] Huang B., Chang J. Y., Li B. W., He L., Liang R. S., He H., Miao A. J., Li H. (2025). An effective
method for distinguishing
extracellular and intracellular nanoparticles through chemical extractions. Aquat. Toxicol..

[45] Zhao J., Bertoglio B. A., Devinney Jr M. J., Dineley K. E., Kay A. R. (2009). The interaction
of biological and noxious transition metals with the zinc probes FluoZin-3
and Newport Green. Anal. Biochem..

[46] Thomas D. G., Smith J. N., Thrall B. D., Baer D. R., Jolley H., Munusamy P., Kodali V., Demokritou P., Cohen J., Teeguarden J. G. (2018). ISD3: a
particokinetic model for
predicting the combined effects of particle sedimentation, diffusion
and dissolution on cellular dosimetry for in vitro systems. Part. Fibre Toxicol..

