# Supplementary material for: Cellular Uptake, Intracellular Fate, and Cytotoxicity of Micro- and Nanoparticulate Nickel Substances in Human Lung Cells
Source: Chem Res Toxicol. 2026 Aug 4;39(8):1532–45. doi: 10.1021/acs.chemrestox.6c00074 (PMC13488609; doi:10.1021/acs.chemrestox.6c00074)
Supplement: Supplementary file 1 [file tx6c00074_si_001.pdf]

Supporting Information

Cellular Uptake, Intracellular Fate and Cytotoxicity of Micro- and Nanoparticulate Nickel Substances in Human Lung Cells

Hanna L. Karlsson*[1,2], Abishek Arora[1], Gunilla Herting[3], Luisa Path[1], Samuel Buxton[4], Tara Lyons-Darden[4], and Inger Odnevall[3,5]

[1]Institute of Environmental Medicine, Karolinska Institute, SE-17177 Stockholm, Sweden

[2] Department of Pharmaceutical Biosciences, Uppsala University, SE-751 24 Uppsala, Sweden

[3]KTH Royal Institute of Technology, School of Engineering Sciences in Chemistry, Biotechnology and Health, Department of Chemistry, Division of Surface and Corrosion Science, SE-100 44 Stockholm, Sweden

[4]NiPERA Inc., 2525 Meridian Parkway, Suite 240, Durham, NC 27713, USA

[5]AIMES–Center for the Advancement of Integrated Medical and Engineering Sciences at Karolinska Institute, SE-100 44 Stockholm, Sweden

* Corresponding author: Hanna.L.Karlsson@ki.se, Hanna.L.Karlsson@uu.se

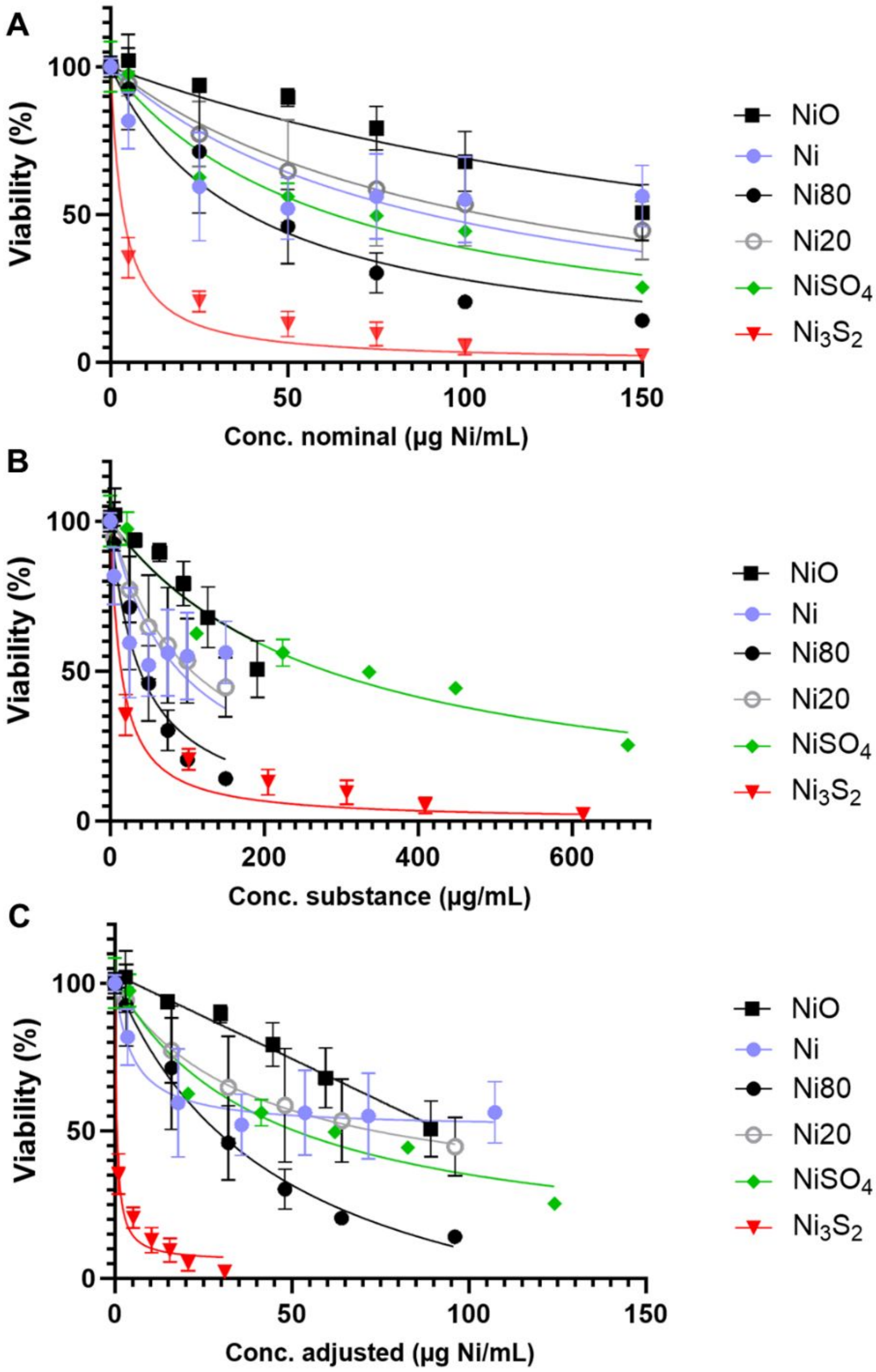

Fig S1. Dose-response of cytotoxicity when considering the nominal dose as µg Ni/mL (A), the nominal dose as µg substance/mL (B) and the µg Ni/mL calculated added dose (C) (i.e. adjusted), based on separate experiment exploring dose transfer. The data show the cytotoxicity at 48 h.

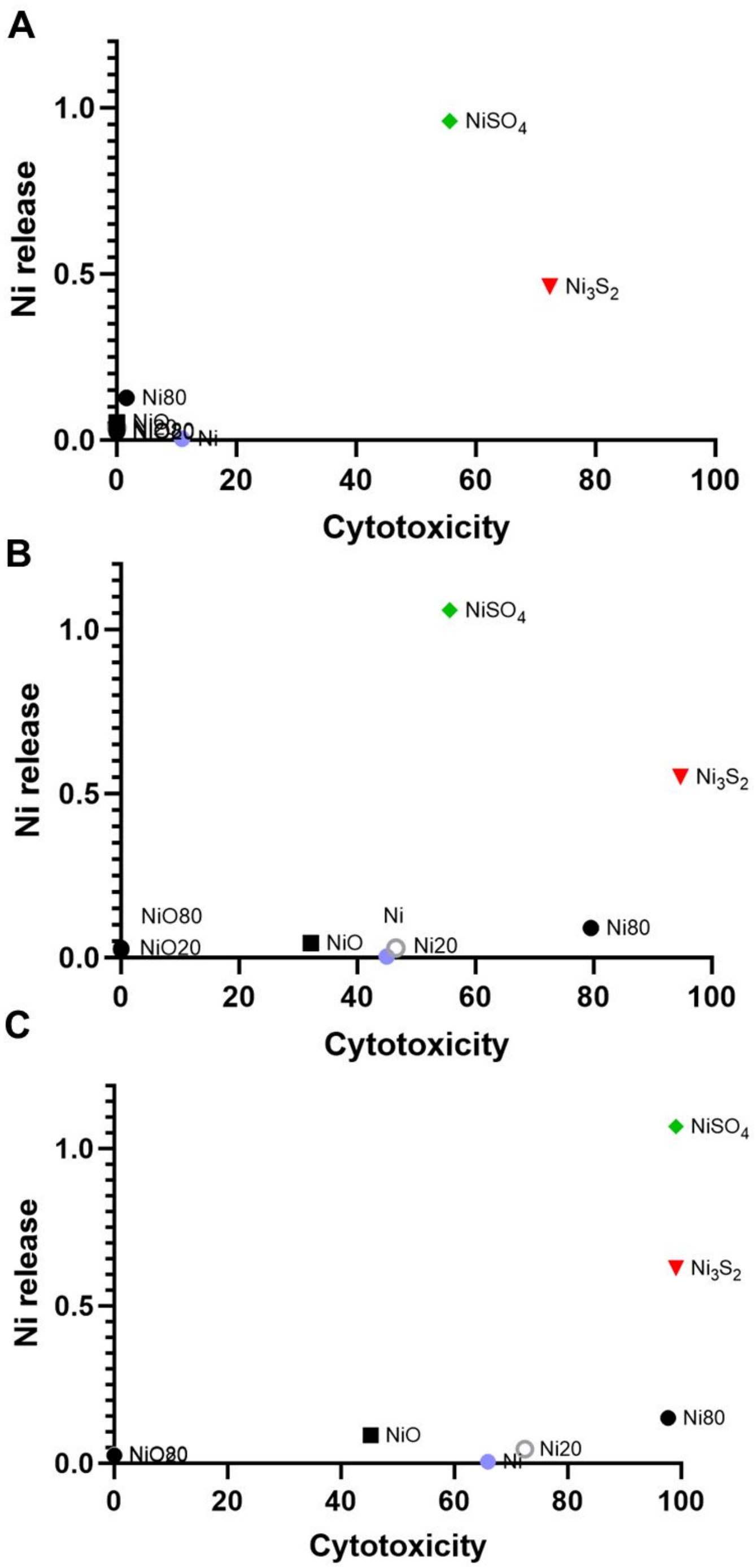

Fig. S2. Correlation between biodissolution (Ni release) in cell media and cytotoxicity at 100 µg/mL (Ni nominal dose) at 24 h (A), 48 h (B) and 72 h (C). There was a significant correlation between cytotoxicity at 100 µg/mL and Ni release at 72 h (Spearman's rank correlation, $r = 0.8$, $p < 0.05$), whereas no such relationship was observed at 24 or 48 h.

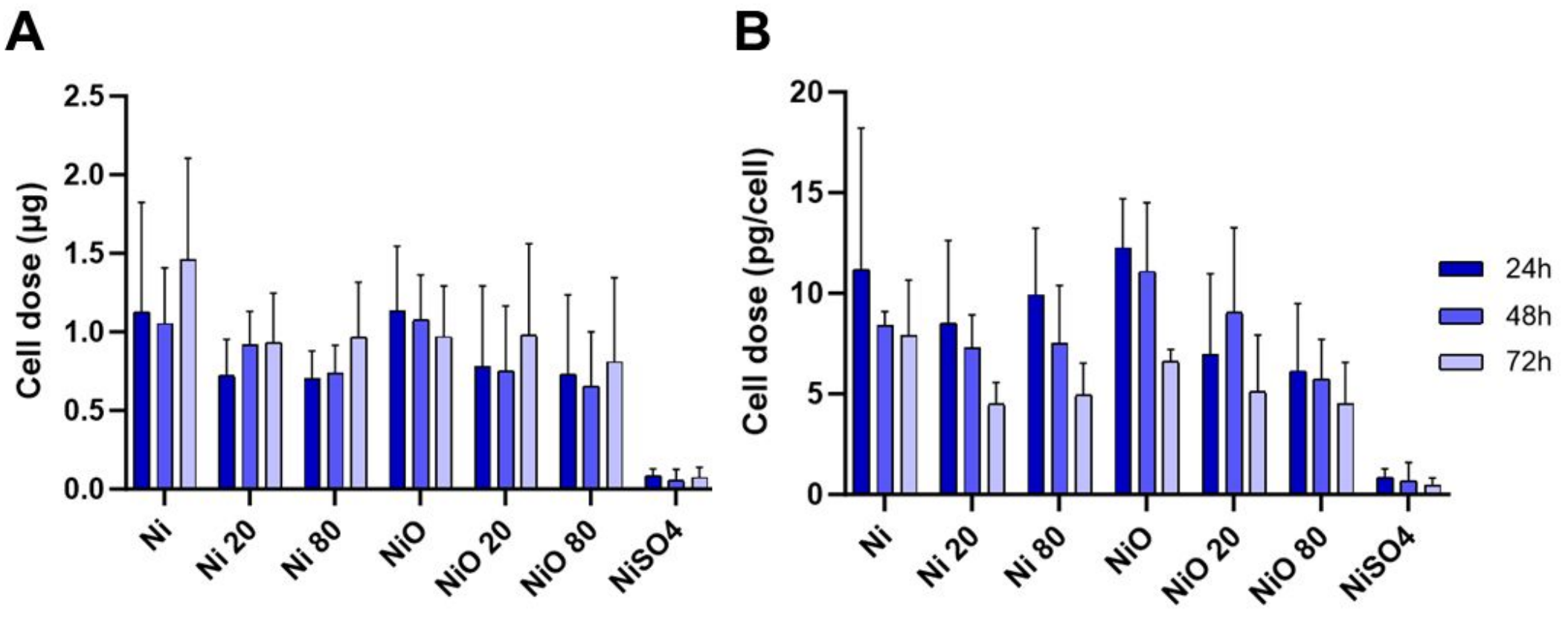

Fig. S3. Uptake of various Ni substances as well as soluble Ni ($NiSO_4 \cdot 6H_2O$) in BEAS-2B cells after different exposure times shown as A) total mass (µg Ni) in the cells as well as B) mass (pg Ni) per cell.

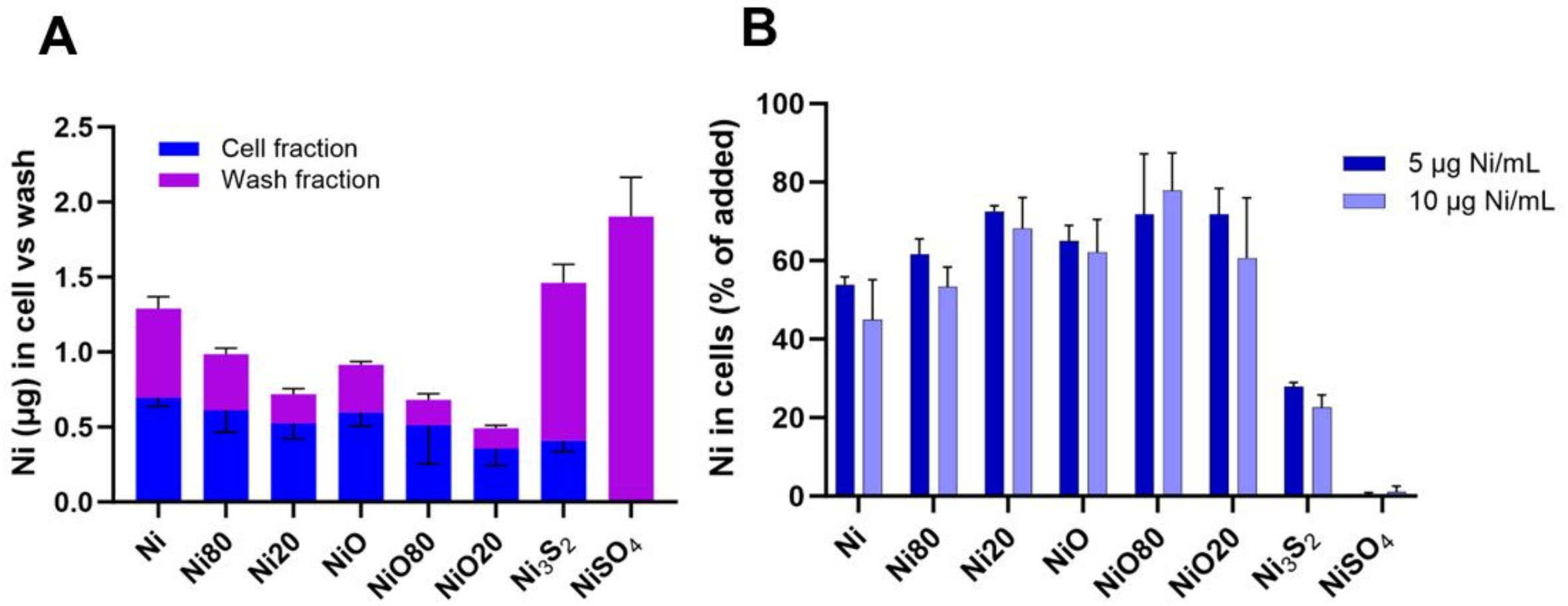

Fig. S4. Uptake of various Ni substances in BEAS-2B cells after 24 h. The total Ni uptake was measured in both the cell fraction and wash fraction (5 µg Ni/mL, 2.5 µg Ni added) (A) and the extent (%) of Ni observed in the cell fraction compared to the total added (wash + cells) Ni content was calculated for both 5 µg Ni/mL (2.5 µg Ni added) and 10 µg Ni/mL (5 µg Ni added) (B). The bars show the mean + SD (n=3).

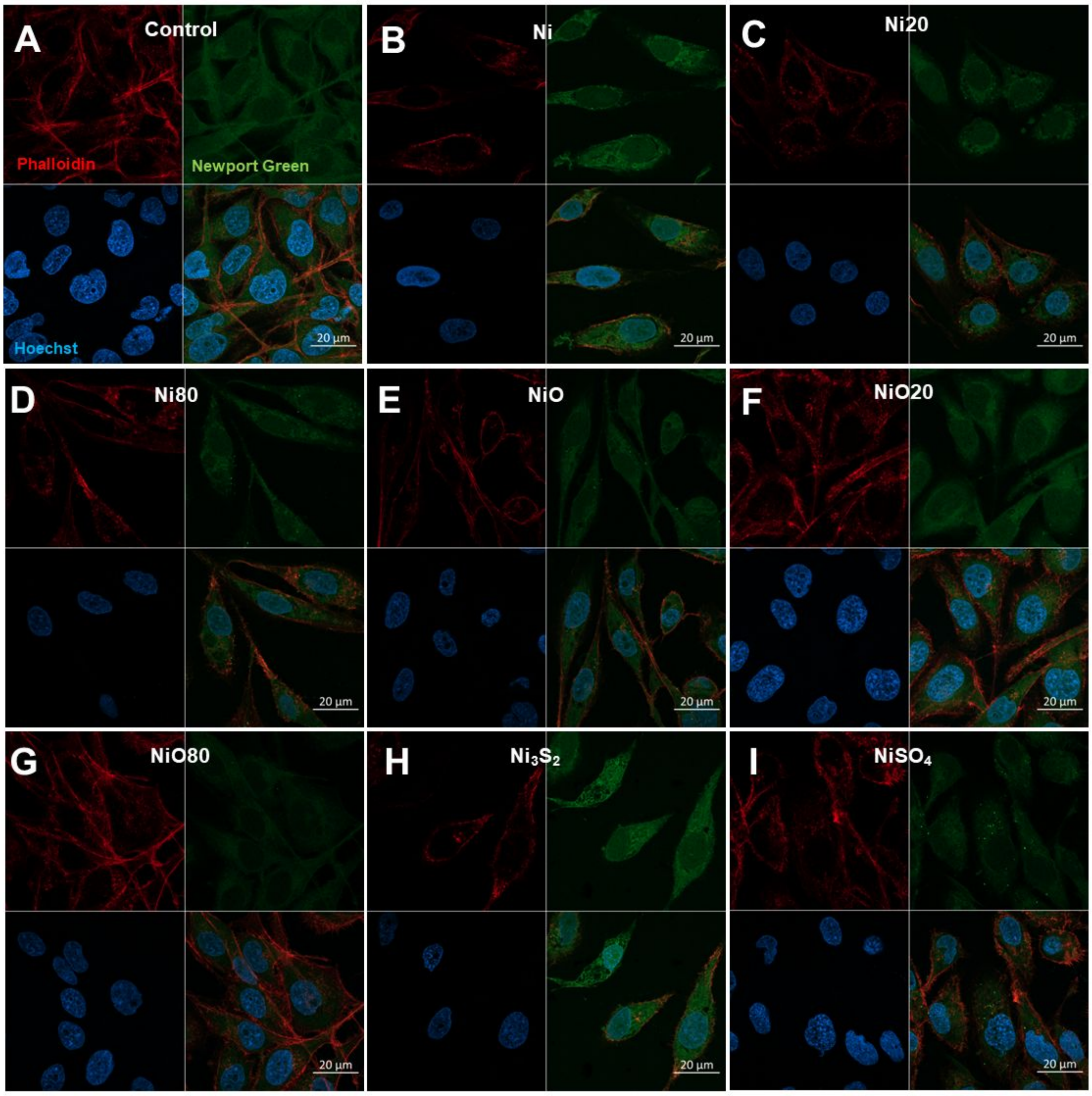

Fig. S5. Newport Green distribution in BEAS-2B cells exposed for 24 h to the various Ni substances in concentrations of 50 µg Ni/mL. Each image presents the cell morphology and cytoskeletal structure in quadrant 1 (Phalloidin - red), the nucleus in quadrant 2 (Hoechst-blue), the presence of intracellular Ni^{2+} ions in quadrant 3 (Newport Green-green) and the overlay image in quadrant 4. The figure includes images from untreated controls (A), and after exposure to Ni MPs (B), Ni20 NPs (C), Ni80 NPs (D), NiO MPs (E), NiO20 NPs (F), NiO80 NPs (G), Ni_3S_2 (H) and $NiSO_4 \cdot 6H_2O$ (I). The scale bars indicate 20 µm.
